# Single-Cell Profiling Identifies Reward Behavior-Related Neurons and Alterations in the Ventral Tegmental Area Based on *Arvcf*-Knockout Mouse Model

**DOI:** 10.34133/research.1030

**Published:** 2025-12-10

**Authors:** Meidi Zhang, Bin Zhang, Yan Wang, Qiaoning Chang, Jingmin He, Zhongli Yang, Ming D. Li

**Affiliations:** ^1^ State Key Laboratory for Diagnosis and Treatment of Infectious Diseases, National Clinical Research Center for Infectious Diseases, National Medical Center for Infectious Diseases, Collaborative Innovation Center for Diagnosis and Treatment of Infectious Diseases, The First Affiliated Hospital, Zhejiang University School of Medicine, Hangzhou 310009, China.; ^2^ College of Animal Sciences, Shanxi Agricultural University, Taigu, Shanxi, China.; ^3^ College of Life Sciences, Shanxi Agricultural University, Taigu, Shanxi, China.; ^4^ Research Center for Air Pollution and Health, Zhejiang University, Hangzhou 310058, China.

## Abstract

The ventral tegmental area (VTA) is a crucial brain region for dopamine synthesis and reward processing, yet the molecular diversity and functional roles of nondopaminergic VTA neurons remain poorly characterized. While *ARVCF* (a member of ARVCF delta catenin family) has been implicated in dopamine release and reward-related behaviors (e.g., nicotine/alcohol and natural rewards), its influence on VTA neuronal subpopulations at single-cell resolution is unknown. Based on the *Arvcf*-knockout (*Arvcf*-KO) mouse model and nicotine exposure, we constructed a reward behavior-related profile at the single-cell transcriptome level and explored the cell subpopulations associated with reward behavior in VTA, as well as how these populations communicate with dopaminergic neurons. Following single-nucleus RNA sequencing (snRNA-seq) from VTA, we obtained 96,240 cells of wild-type and *Arvcf*-KO mice with and without nicotine treatment. Subsequent cell-type abundance analysis revealed a significant reduction in the abundance of neuronal populations upon *Arvcf*-KO (FDR < 0.05). By integrating single-cell transcriptomics, neurology proteomics, and multiplex immunofluorescence imaging, we discovered a subpopulation of glutamatergic–dopaminergic combinatorial neurons, which is significantly associated with reward. Further cell communication analysis indicated that *Arvcf*-KO reduced the signal output from these neurons to dopaminergic neurons, represented by Wnt signaling. Finally, integrative analyses of metabolite detection and 2-way analysis of variance based on snRNA-seq indicated that the glutamatergic properties represent a key regulator of reward learning behaviors induced by nicotine and related stimuli. Taken together, our single-cell analysis identified that VTA combinatorial neurons are crucial for nicotine-induced reward through cellular signaling and glutamatergic properties, implying potential new therapeutic targets for addiction treatment.

## Introduction

The ventral tegmental area (VTA) has long been investigated as the main brain region involved in reward processing [1,2]. The transmission of dopamine (DA) from VTA to the nucleus accumbens (NAc) and other limbic regions not only plays a crucial role in the processes underlying reward learning and behavioral reinforcement, but also serves as a pivotal structure that triggers addiction and various neuropsychiatric diseases [3–5]. The release of DA from neurons within VTA possesses sufficient capability to support positive reinforcement and reward learning [6,7]. Consequently, DA neurotransmission is commonly regarded as a fundamental signal of reward-related behavioral condition [8]. However, there is increasing evidence for the essential functional and physiological roles of glutamatergic and γ-aminobutyric acid (GABA)ergic neurotransmission in reward-related behaviors [9,10].

Optogenetic studies have shown that VTA GABAergic neurons can respond to reward-predicting cues and aversive stimulus [11,12]. In contrast to dopaminergic neurons, activating GABAergic neurons is sufficient to inhibit reward consumption or induce avoidance behavior [13,14]. Except for the GABAergic and DA neurons concentrated in the lateral region of VTA, there is also a large number of glutamatergic neurons (mainly expressing VGLUT2 mRNA) concentrated in the medial region of VTA [15,16]. Glutamatergic neurons in this region outnumber tyrosine hydroxylase (*TH*^+^) dopaminergic neurons and emit substantial projections to the prefrontal cortex and NAc [15–17]. Recent studies have demonstrated that activation of VTA glutamatergic neurons can reinforce operant behavior and lead to DA release via corelease of glutamate neurotransmitters and/or local excitatory synapses onto VTA dopaminergic neurons [18–20]. Meanwhile, parallel evidence suggested that the presence of “combinatorial” neurons that coexpress genes taking part in the synthesis and/or transport of multiple neurotransmitters can corelease multiple neurotransmitters [1,18,19,21,22]. Notably, the axons of VTA dopaminergic neurons that project to the NAc can corelease glutamate and DA, employing independent vesicle pools for each neurotransmitter [23]. The presence of different types of neurons in the VTA with corelease of neurotransmitters increases the factual complexity and difficulty of the corresponding research.

Our previous work has shown that the *ARVCF* gene (a member of ARVCF delta catenin family), which can regulate cadherin level [24] and promote proliferation and synaptic differentiation of ventral midbrain dopaminergic progenitor [25,26], was significantly associated with nicotine and alcohol dependence through human genome-wide association studies [27,28]. Further animal behavior studies have demonstrated the deficiency of *Arvcf* impaired learning capacity and rewarding response to drug reward (nicotine or alcohol) [27–29]. Additionally, fiber photometry analysis demonstrated the promoting role of VTA dopaminergic *Arvcf* in regulating DA synthesis and release in the VTA–NAc circuit through nicotine, alcohol, and natural reward (food/drink/social investigation) stimuli [28,29]. Even so, due to the complex composition and heterogeneity of VTA neurons, further research is needed on how *Arvcf* regulates DA synthesis and release, especially at a high-resolution level. Based on the above information, we were interested in studying how *Arvcf* gene impacts and mediates reward behavior in the VTA region. However, previous reported studies on reward learning in the VTA region have often relied on low-throughput methods [30,31], which could not definitively explore the reward-related cell atlas within this brain subregion. The recent advances in single-cell sequencing technology have enabled the collection of the whole transcriptomes from numerous cells within VTA, which circumvents previous technological limitations.

On the basis of a 7-day nicotine treatment protocol validated by the conditional place preference (CPP) test [27], in this report, we performed single-nucleus RNA sequencing (snRNA-seq) on 96,240 VTA nuclei from the wild-type (WT) and *Arvcf*-knockout (*Arvcf*-KO) mice with and without nicotine treatment. Our results not only confirmed the presence of combinatorial neurons, but also identified the reward learning-related neuron subpopulation by comparing the transcriptome profiles of the *Arvcf*-KO group and WT group. We further revealed the transcriptional regulation mechanisms by which the reward-related neurons interact with dopaminergic neurons in the VTA region. Finally, we discovered the dynamic changes in transcriptional characteristics and functions of these neurons following nicotine treatment. Together, this study provides a new perspective for studying the complex molecular mechanisms of reward learning behaviors.

## Results

### *Arvcf*-KO predominantly impairs neuronal populations in mice VTA region revealed by the snRNA-seq technique

**Fig. 1.**
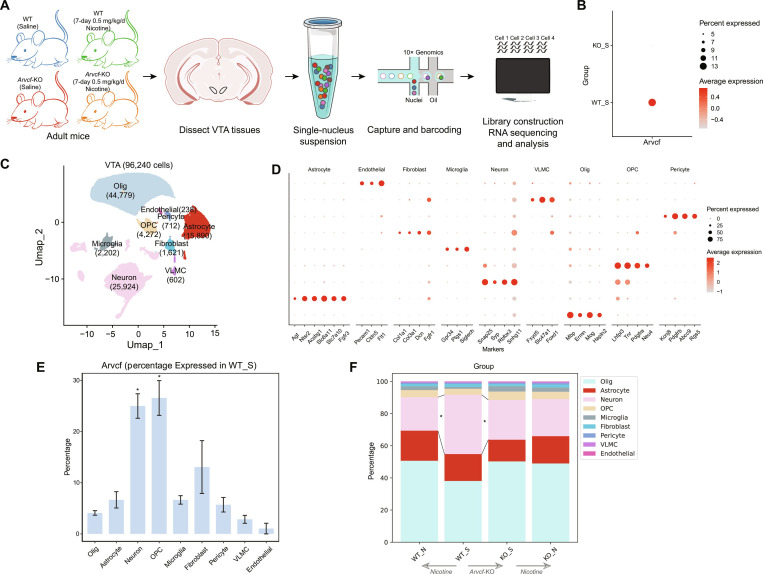
Transcriptional atlas of cell types in the VTA of WT and *Arvcf*-KO mice. (A) snRNA-seq workflow. Tissue was harvested from WT mice with saline control (WT_S) or nicotine-treated (WT_N), *Arvcf*-KO mice with saline control (KO_S) or nicotine-treated (KO_N) (*n* = 3/group) prior to nuclei isolation and snRNA-seq (created in BioRender). (B) Dot plot displays *Arvcf* expression level of the WT_S group and the KO_S group across all cells. (C) Uniform manifold approximation and projection (UMAP) of 9 major cell types in the mice VTA. (D) Dot plot of major marker genes for identified cell clusters. (E) Error bar plot shows the proportion of *Arvcf*-expressing cells in each cell type for WT_S samples. Data are presented as mean ± SD (*P < 0.05). (F) Percentage (*y*-axis) of each cell type (color legend) for each group (*x*-axis). *Significance at an FDR level of 0.05, calculated by scCODA.

To investigate the cell landscape by which *Arvcf* affects reward behavior, we conducted snRNA-seq analysis in the VTA region of WT mice and *Arvcf*-KO mice with 7-day or without nicotine treatment (Fig. 1A and Fig. S1). At the same time, we assessed the expression level of *Arvcf* in the single-cell transcriptome of WT samples with saline-control (WT_S), and *Arvcf*-KO samples with saline-control (KO_S), confirming the technical success and quality of the data (Fig. 1B). Following quality control and unsupervised clustering, we obtained 9 major cell types in 96,240 individual VTA nuclei (Fig. 1C and D, Fig. S2, and Table S1). Because susceptibility genes generally tend to be more selectively expressed in pathogenic cells, we first identified a certain specificity of *Arvcf* in these identified cell populations in the WT_S group. As shown in Fig. 1E, *Arvcf* was widely expressed in neurons as well as in oligodendrocyte precursor cells. Subsequently, we examined the effects of *Arvcf*-KO and nicotine treatment on cellular composition, respectively (Fig. 1F). A significant reduction in the neuronal populations was observed in the KO_S group compared to the WT_S group. Whereas nicotine treatment also significantly decreased the neuronal proportion in WT mice, it had no significant effect on any cell population in *Arvcf*-KO mice (false discovery rate [FDR] < 0.05; Fig. S3 and Table S2). Based on these snRNA-seq results, we concluded that *Arvcf*-KO mainly affected the neuronal populations in the VTA region; thus, we decided to focus on neuronal populations in this study.

### Integration of proteomics reveals neuronal subpopulation involved in reward learning at single-cell resolution

**Fig. 2.**
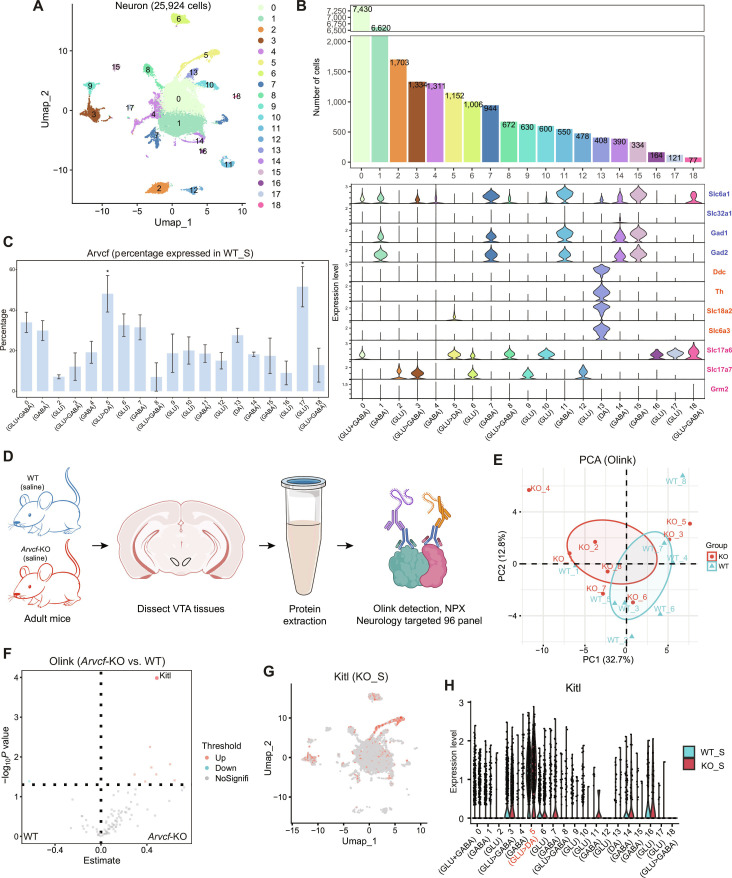
Multi-omics analysis of neuronal transcription and neurology-related proteins in the VTA of WT and *Arvcf*-KO mice. (A) UMAP of 19 neuronal subclusters in the VTA with resolution of 0.2. (B) Bar graph displaying number of cells per neuronal cluster (top) and violin plot for genes involved in the synthesis and transport of GABA, DA, and glutamate (bottom). (C) Error bar plot shows the proportion of *Arvcf*-expressing cells in each cell type for WT_S samples. Data are presented as mean ± SD (*P < 0.05). (D) Workflow for the Olink Target 96 Neurology panel. Tissue was harvested from control-treated WT mice and *Arvcf*-KO mice (*n* = 8/group) prior to protein extraction and Olink detection (created in BioRender). (E) PCA with the 95% confidence ellipse between the WT group and Arvcf-KO group based on a total of 92 proteins. (F) Volcanic map showing the DAPs between the *Arvcf*-KO group and WT group. Top proteins with adjusted *P* value are labeled. (G) UMAP plot displays transcriptional expression level of *Kitl* in KO_S neuronal populations. (H) Violin plot of *Kitl* transcriptional expression level across neuronal subpopulations in WT_S and KO_S groups.

To explore neuronal populations more thoroughly, we reclustered all neurons, which revealed 19 transcriptionally distinct cell populations at a resolution of 0.2 determined by modularity *Q* value and area under curve (AUC) method (Fig. 2A and B, Fig. S4, and Table S3). Since VTA and substantia nigra (SN) are the 2 main DA-producing areas with different functions, we first determined if our VTA subsections contained the nearby SN structures and found that the DA neurons (Cluster 13) rarely coexpressed *Sox6* and *Aldh1a1*; a coexpression of these 2 markers is an indication of the presence of SN structure (Fig. S6B) [32,33]. Furthermore, we found that some DA neurons were similar to *Slc17a6*^+^/*Otx2*^+^ or *Vip*^+^/*Gipr*^+^ populations previously described in VTA (Fig. S6A) [21,32]. Meanwhile, a low DA/GLU or DA/GABA ratio in the WT_S group was similar to previous single-cell transcriptomes research (Table S4) [21,34]. To further verify the anatomical purity of the VTA, we examined the expression levels of markers (*Pvalb*, *Sst*, and *Slc17a8*) in its adjacent region, the interpeduncular nucleus (IPN) (Fig. S6C) [35,36], and found that the expression proportion of these markers was less than 5%, supporting the high purity of the VTA samples obtained in our dissection.

In addition to typical neurons including dopaminergic, glutamatergic, and GABAergic neurons, we also identified combinatorial neuron populations, which may synthesize and/or release more than one neurotransmitter (Clusters 0, 3, 5, 8, and 18) (Fig. 1B) as found in other reports [1,22,37]. To provide direct evidence for the presence of combinatorial neurons, we examined the overall coexpression patterns by analyzing the proportion of cells that coexpress unique genes involved in the synthesis, transport, or release of GABA, glutamate, or DA among each cluster (Fig. S5). We observed that the 3 GLU>GABA neuronal clusters (i.e., Clusters 3, 8, and 18) even had different coexpression patterns (Fig. S5A to C). Furthermore, we found that there was no aggregated high expression of *Grm2* (a group II metabotropic glutamate receptor) in neuronal clusters, but with higher percentage of expression only in Clusters 3 and 8 (GLU>GABA) (Fig. 2B and Figs. S5A and B and S6D). Interestingly, we found that *Grm5* involved in reward- and nicotine dependence (ND)-related behaviors [38,39] was widely expressed in all types of neurons examined in this study.

Next, we explored the impact of *Arvcf* on neurons at the transcriptome level with the goal of finding relevant neuronal cell populations in which it participates in reward learning. We first assessed the expression percentage of *Arvcf* per neuronal cluster in the WT_S group and found that *Arvcf* was more abundant in Clusters 5 (GLU>DA) and 17 (GLU) than other neuronal clusters (Fig. 2C). Meanwhile, we employed Olink Target 96 Neurology panel proteomics technology to conduct an in-depth analysis of the differences in neural specific expression patterns between the WT_S group and the KO_S group at the protein level (Fig. 2D). The principal component analysis (PCA) revealed that *Arvcf*-KO induced distinct alterations in the neurology-related protein profile (Fig. 2E). Subsequently, we identified differentially abundant proteins (DAPs) between the KO_S group and the WT_S group, among which Kitl emerged as the most statistically significant (*P* = 0.0001; Fig. 2F and Table S5). To further explore its relevance in a neuronal context, we examined the single-cell transcriptomic data and found that, across all neuronal clusters, *Kitl* was highly expressed specifically in Cluster 5 of the KO_S group and exhibited higher expression compared to the WT_S group (Fig. 2G and H). Therefore, we concluded that the knockout of *Arvcf* primarily acted on this neuron subpopulation.

### *Arvcf*-KO damages glutamatergic characters of reward learning-related neuronal subpopulation

**Fig. 3.**
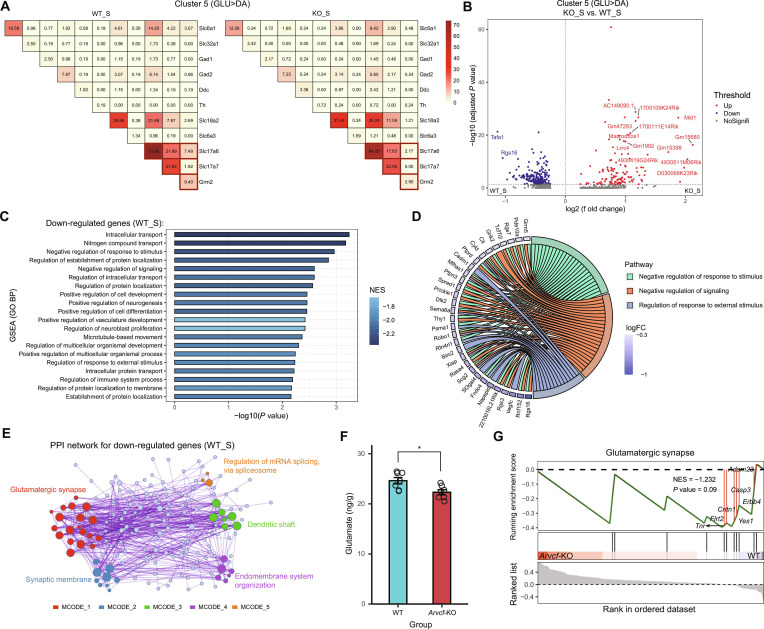
Comparative analysis of VTA GLU>DA neurons between the KO_S group and the WT_S group. (A) Heatmaps depicting proportion of cells coexpressing the gene involved in synthesis, release, or transport of GABA, dopamine, and glutamate neurotransmitters within neuronal Cluster 5 (GLU>DA) (WT_S vs. KO_S). (B) Volcanic map showing the DEGs between the KO_S group and the WT_S group in Cluster 5. Top genes with adjusted *P* value are labeled. (C) Bar plot indicating the top 20 GO BP terms by GSEA between the WT_S group and the KO_S group in Cluster 5. (D) Chord diagram showing the core genes of the pathways of response and feedback. (E) Protein–protein interaction network using the down-regulated DEGs of Fig. 2C, including 5 MCODE networks. Each color represents an MCODE network. Gene Ontology enrichment analysis was applied to each MCODE network to extract “biological meanings” from the network component, and the top terms (with the smallest *P* values) were retained. (F) Glutamate neurotransmitter level in the VTA of mice was analyzed using a liquid chromatograph mass spectrometer (LC-MS) (*n* = 7/group). (G) GSEA of Olink Target 96 Neurology panel proteins showing enriched in the “Glutamatergic Synapse” pathway between *Arvcf*-KO and WT mice.

To explore the impact of *Arvcf*-KO on the gene expression profiles of Cluster 5, we analyzed the proportion of cells that coexpress unique genes involved in the synthesis, transport, and/or release of GABA, glutamate, or DA in the respective WT_S and KO_S groups. We found that the KO_S group mainly exhibited a lower proportion of coexpression of glutamatergic markers (*Slc17a6*, *Slc17a7*, and *Grm2*) than the WT_S group (*P* = 0.003762; Fig. 3A). Then, we calculated the differentially expressed genes (DEGs) including 128 up- and 208 down-regulated between the 2 groups and performed Gene Ontology biological processes (GO BP) analysis with gene set enrichment analysis (GSEA) on these identified DEGs (Fig. 3B and C and Table S6). The identified top 20 GO BP terms showed that the WT_S group was mainly enriched in the pathways of molecular and cellular processes (e.g., intracellular transport, nitrogen compound transport, and regulation of establishment of protein localization), response and feedback (e.g., negative regulation of response to stimulus, negative regulation of signaling, and regulation of response to external stimulus), and cell development and regulation (e.g., positive regulation of cell development, neurogenesis, and cell differentiation). As shown in Fig. 3D, we also found that many down-regulated DEGs contributed to the pathways of response and feedback, which may play critical roles in cellular signaling and reward learning. Of these, 9 genes were commonly enriched in all pathways. *Scg2*, *Robo1*, *Sema6a*, and *Mfhas1* are involved in the regulation of neuronal and nervous system functions; *Xiap* and *Cyld* are involved in cell development and apoptosis. Moreover, a protein–protein interaction (PPI) network was enriched for the down-regulated DEGs, resulting in 5 Molecular Complex Detection (MCODE) networks (Fig. 3E). The “glutamatergic synapse”, “synaptic membrane”, “dendritic shaft”, “endomembrane system organization”, and “regulation of mRNA splicing, via spliceosome” terms were enriched in this PPI network (Table S7). We further detected that the VTA region of *Arvcf*-KO mice had less glutamate neurotransmitters than that of WT mice, which may be influenced by Cluster 5 neurons (*P* = 0.0149; Fig. 3F and Table S8). Additionally, proteomic GSEA also revealed that the WT_S group was enriched in the pathway of glutamatergic synapse (normalized enrichment score [NES] = –1.232, *P* = 0.09; Fig. 3G).

### Differential signaling from reward learning-related neuronal subpopulation to dopaminergic neurons in WT versus *Arvcf*-KO mice

**Fig. 4.**
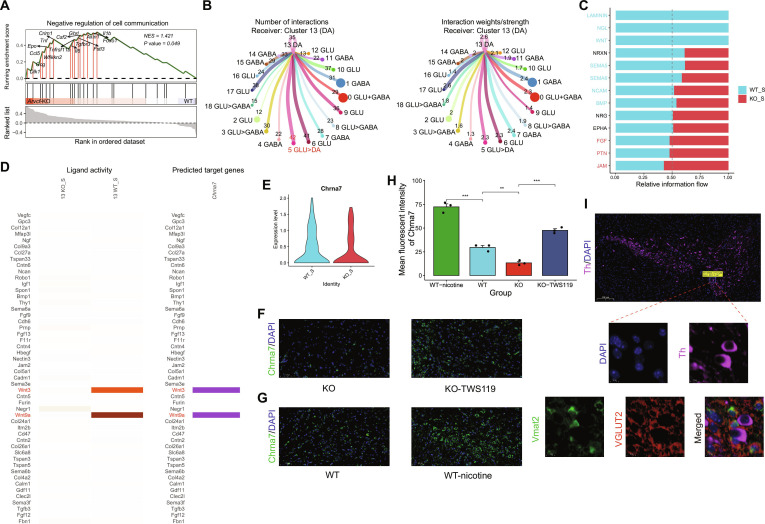
Cellular communication between GLU>DA neurons and DA neurons. (A) GSEA of Olink Target 96 Neurology panel proteins showing enriched in the “Negative Regulation of Cell Communication” pathway between *Arvcf*-KO and WT mice. (B) Circos plots showing the number and strength of incoming signals from sender neuron subpopulations to DA neurons (receiver) in the WT_S group, predicted by CellChat. (C) Bar plots displaying the relative information flow of conservation and specificity pathways from neuronal Cluster 5 (sender) to DA neurons (receiver) between WT_S and KO_S groups. (D) The activity of top 50 ligands and predicted target genes by NicheNet. (E) The gene expression level of predicted target genes in receiver cells (DA neurons). (F) Representative images of immunofluorescent staining of Chrna7 (green) in VTA of *Arvcf*-KO mice under control condition (left) and following Wnt activator TWS119 (right) (*n* = 3/group). Scale bar, 100 μm. (G) Representative images of immunofluorescent staining of Chrna7 (green) in VTA of WT mice under control conditions (left) and following nicotine treatment (right) (*n* = 3/group). Scale bar, 100 μm. (H) Bar plot showing mean fluorescent intensity of Chrna7 in VTA of WT mice under control conditions and following nicotine treatment, *Arvcf*-KO mice under control conditions and following Wnt activator TWS119 (*n* = 3/group). **P < 0.01, ***P < 0.001. (I) Classic coronal view of the VTA illustrated by representative images of immunofluorescent staining of Th (pink) in VTA of WT mice (*n* = 3). Scale bar, 200 μm; Multiple immunofluorescence staining of medical VTA. The staining includes DAPI (blue), Th (pink), Vmat2 (green), and VGLUT2 (red). Magnified scale bar, 10 μm.

Considering that only the relative differences on the basis of single-cell transcriptomics and neurology proteomics have been emphasized, some potential biologically significant changes might have been missed. Thus, besides Cluster 5, we also performed differential expression and functional enrichment analysis for other neuronal subpopulations between the KO_S group and the WT_S group. Of all neuron clusters, only Cluster 13 (i.e., DA) was significantly enriched in stimulus response-related pathways along with Cluster 5 (GLU>DA) (Table 1), where a stimulus–reward association had previously been demonstrated [40,41]. Importantly, these pathways are closely linked to cellular signaling processes. In line with this, proteomic GSEA further showed that the KO_S group was significantly enriched in the negative regulation of cell communication (NES = 1.421, *P* = 0.049; Fig. 4A). Considering that our previous works have also found that *Arvcf*-KO can directly affect DA release under reward stimulation [28,29], then we decided to only focus on the signal communication to dopaminergic neurons. Further CellChat analysis revealed that neurons in Cluster 5 signaled to DA neurons with the highest number and stronger cellular ligand–receptor interactions by comparing with other neurons in the WT_S group (Fig. 4B). Thus, we inferred that the Cluster 5 neurons might send signals to DA neurons and even affect DA release.

**Table 1.** The intersected GO terms in neuronal Clusters 5 (GLU>DA) and 13 (DA). NES indicates normalized enrichment score by GSEA enrichment between the KO_S group and the WT_S group within the corresponding neuronal cluster.

GO term	Cluster 5		Cluster 13	
	*P* value	NES	*P* value	NES
Cellular response to external stimulus	0.0109	−1.6203	0.0016	−2.1505
Cellular response to extracellular stimulus	0.0109	−1.6203	0.0003	−2.3504
Response to extracellular stimulus	0.0388	−1.5652	0.0127	−1.714

To better understand how Cluster 5 neurons influence DA neurons, we then focused on the signaling differences caused by *Arvcf*-KO from Cluster 5 (GLU>DA) to Cluster 13 (DA). We first compared cell–cell communication strength and probability (i.e., information flow) in Clusters 5 and 13 to identify conserved and specific signaling pathways between the WT_S group and the KO_S group (Fig. 3C). The results showed that the WT_S group exhibited stronger overall signaling activity compared to the KO_S group. Notably, pathways involving Laminin, NGL (netrin-G ligand), and Wnt were all uniquely active in the WT_S group. Further, we investigated ligand–receptor interactions and predicted the different impacts of the niche on DA neurons between the 2 groups. Differential NicheNet analysis inferred that the expression of *Wnt3* and *Wnt9a* as ligands were down-regulated and actively inhibited in the KO_S group (Fig. 4D and Fig. S7). Through inhibiting Wnt signaling pathway mediated by these genes, *Chrna7* was predicted as the target gene in DA neurons and down-regulated by *Arvcf*-KO (Fig. 4D and E). To validate the association between the Wnt pathway and *Chrna7*, we administered the Wnt/β-catenin pathway activator TWS119 to *Arvcf*-KO mice for 14 days [42]. Immunofluorescence analysis revealed an increase in Chrna7 protein levels in the VTA following Wnt activator treatment (*P* = 0.00015; Fig. 4F and H). Furthermore, we confirmed that the *Arvcf*-KO indeed down-regulated Chrna7 expression in VTA at the protein level (*P* = 0.0043). Additionally, nicotine treatment resulted in higher Chrna7 protein levels in the VTA of WT mice (*P* = 0.0008; Fig. 4G).

Based on the above results, we sought to validate neuronal Cluster 5 and explore its spatial context through multiplex immunofluorescence. Staining for the defining markers Slc17a6 (VGLUT2) and Slc18a2 (Vmat2) successfully identified a subpopulation of double-positive cells within the medial VTA, as previously discovered [43] (Fig. 4I). As part of an initial spatial assessment, we noted that some of these double-positive neurons appeared to reside in close proximity to Th-positive dopaminergic neurons.

### The effects of nicotine stimulation on the *Slc17a6*+ glutamatergic neuronal subpopulations

**Fig. 5.**
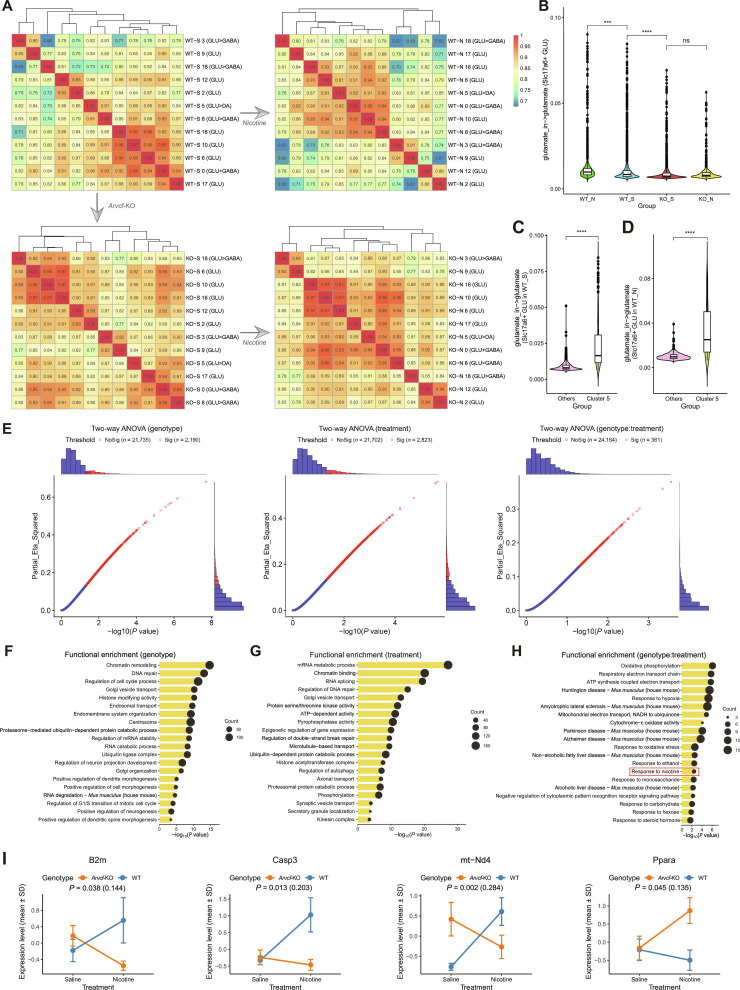
The comparative analysis of *Slc17a6*+ GLU neurons following nicotine treatment. (A) Heatmaps indicate that Pearson’s correlation among all GLU neuronal clusters based on whole transcript counts in WT_S, KO_S, WT_N, and KO_N groups. (B) Violin plot of scFEA scores reflecting glutamate metabolic flux of *Slc17a6*+ GLU neurons (Clusters 0, 5, 6, 8, 10, 16, 17, and 18) in WT_S, KO_S, WT_N, and KO_N groups. (C and D) Comparison of scFEA scores between Cluster 5 and other *Slc17a6*+ GLU neurons within the WT_S group and the WT_N group, respectively. ***P < 0.001, ****P <0.0001; ns, no significance. (E) Scatter plots with histograms showing 2-way ANOVA results of genotype (WT vs. *Arvcf*-KO) effect, treatment (Saline vs. Nicotine) effect, and genotype:treatment interaction effect. Red represents significant effect [*x*-axis: Pr(>*F*) < 0.05 and *y*-axis: partial eta squared ≥ 0.06]. (F to H) Line plots displaying functional enrichment results of GO and KEGG terms for genes with significant genotype effect, treatment effect, and genotype:treatment interaction effect (*P* < 0.05). The size of the black dots indicates the number of enriched genes. (I) Error dot plots show the expression level of genes enriched in the “Response to nicotine” pathway by genotype:treatment interaction effect. *P* < 0.05 and the text in parentheses is partial eta squared.

To investigate whether the KO-induced attenuation of glutamatergic signatures was driven specifically by Cluster 5 or represented a broader effect across all glutamatergic neurons, we focused on the 2 major glutamatergic subpopulations: *Slc17a6*+ (*VGLUT2*+) and *Slc17a7*+ (*VGLUT1*+) neurons (Fig. 2B), which are known to possess distinct spatial distributions and functions [44]. By comparing the global transcriptional similarity between these 2 subpopulations under different conditions using a whole-transcriptome correlation analysis, we found that in WT groups (both saline-control and nicotine treatment), the *Slc17a6*+ (Clusters 0, 5, 6, 8, 10, and 16 to 18) and *Slc17a7*+ (Clusters 2, 3, 9, and 12) populations exhibited low transcriptional similarity (Pearson’s correlation coefficient < 0.8; Fig. 5A), consistent with their established heterogeneity. In contrast, these 2 subpopulations showed substantially higher correlation in both KO groups. This finding suggested that *Arvcf*-KO specifically blurs the transcriptional distinction between these 2 glutamatergic lineages. Based on this result and the prior prominence of Cluster 5 (a *Slc17a6*+ cluster), we proposed that *Arvcf*-KO primarily impairs the native state of the classical *Slc17a6*+ glutamatergic subpopulation in the VTA (Fig. S8) [45].

Then, we estimated the flux of glutamate within *Slc17a6*+ glutamatergic neuronal subpopulations (Clusters 0, 5, 6, 8, 10, and 16 to 18) in WT_S, WT_N, KO_S, and KO_N groups (Fig. 5B). Under *Arvcf*-KO conditions, the glutamate metabolic cycling was reduced (*P* < 2.2e−16) and showed no significant changes following nicotine treatment. In contrast, nicotine treatment significantly enhanced the glutamate metabolic cycling in WT mice (*P* = 0.0001191). Interestingly, Cluster 5 consistently exhibited the highest contribution in both the WT_S and WT_N groups (*P* < 2.2e−16, Fig. 5C).

To better assess the effects of genotype (*Arvcf*-KO) and nicotine treatment on Slc17a6+ glutamatergic neuronal populations, we performed a 2-way analysis of variance (ANOVA) (Fig. 5E). This analysis identified a substantial number of genes influenced by each factor: 2,190 genes with a significant main effect of genotype; 2,823 genes with a significant main effect of treatment; 361 genes with a significant interaction effect of genotype:treatment (*P* < 0.05 and partial eta squared > 0.06; Table S9). Functional enrichment analysis was subsequently performed on each of these gene sets to identify associated biological pathways. The genes with significant genotype effect mainly affected the following pathways: DNA repair, regulation of mRNA stability, histone modifying activity, ubiquitin, and positive regulation of neurogenesis (Fig. 5F); the genes with significant treatment effect mainly affected similar functional pathways: regulation of DNA repair, mRNA metabolic process, epigenetic regulation of gene expression, ubiquitin, and axon transport (Fig. 5G). Importantly, the genes exhibiting a significant interaction effect were primarily enriched in pathways related to neurodegenerative diseases—such as Huntington disease, Parkinson disease, and Alzheimer disease—and also influenced functional pathways associated with response to nicotine and ethanol (Fig. 5H). Among the 4 nicotine-responsive genes identified through enrichment analysis, 3 (*B2m*, *Casp3*, and *mt-Nd4*) showed a large effect size (partial eta squared > 0.14), while 1 (*Ppara*) exhibited a medium effect size (partial eta squared > 0.06) (Fig. 5I).

## Discussion

It is generally believed that the DA neurons as the main components of VTA might be attributed to the technical limitations of conventional detection methods such as immunofluorescence or in situ hybridization, which typically capture only localized neuronal subpopulations within tissue sections [15,17]. With these organizational research techniques, numerous studies have clearly demonstrated that the VTA region consists of heterogeneous cell types [1,33,34,46]. However, due to their relatively low throughput and resolution, these methods are unable to adequately resolve and differentiate individual cells across the entire region. Recently, single-cell sequencing approaches have been used to explore the molecular heterogeneity within the region [32–34,47]. So far, the majority of studies were focused on mixed cells from SN and VTA [34], or a subset of cells that have been fluorescently labeled and fluorescence-activated cell sorting (FACS)-isolated from the general midbrain regions [32,33,47]. It is known that the dopaminergic neurons located in SN and VTA are distinct in terms of their functions. SN neurons are crucial elements for movement and motor, while VTA neurons play a pivotal role in emotive and reward learning [48,49]. The limitations of research techniques and their mixing with cells in other brain subregions have led to a lack of comprehensive research on the mechanisms underlying reward learning in the VTA.

**Fig. 6.**
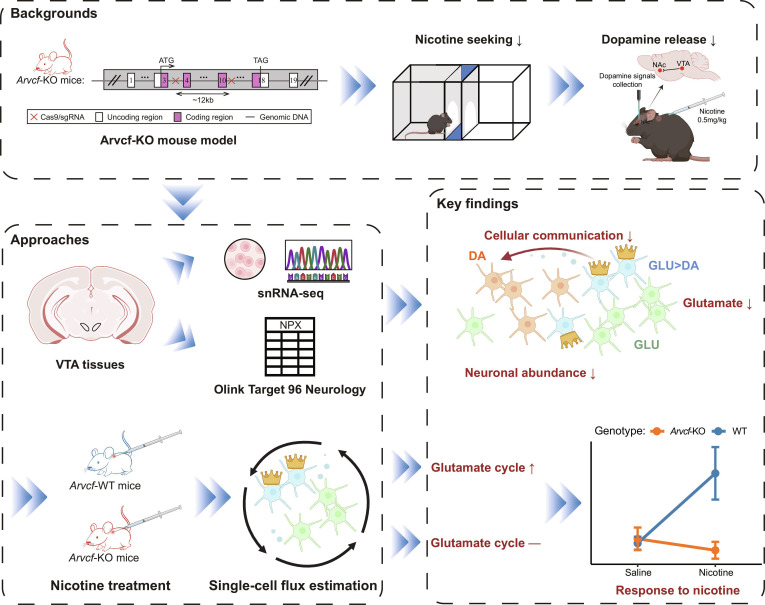
Schematic of impaired neuronal communication and blunted nicotine response in the VTA following *Arvcf* knockout.

It has been reported that *ARVCF* exhibits the capability to modulate cadherin expression levels [24], thereby enhancing the proliferation and synaptic differentiation of dopaminergic progenitor cells in the ventral midbrain [25,26]. Very recently, we have demonstrated the important role of *Arvcf* in nicotine- or alcohol-induced CPP and DA synthesis and release in the VTA–NAc circuit [29], but it is still necessary to further explore how it mediates the regulatory mechanisms underlying DA release and reward behavior at the single-cell level. To attack this issue, we carried out this snRNA-seq analysis study by utilizing the *Arvcf*-KO animal model and nicotine as a reward stimulus. We first identified a subgroup of neurons with transcriptional alterations potentially linked to reward-related behaviors (Fig. 6).

It is generally thought that the mechanisms underlying a disease or an abnormal physiological state of interest might be more related to one or several types of cells [50,51]. Our results showed that *Arvcf* was widely expressed in VTA neurons of WT mice, particularly in Cluster 5 (GLU>DA)—a combinatorial neuronal subpopulation characterized by primary expression of *Slc17a6* (*VGLUT2*) and coexpression of *Slc18a2* (*Vmat2*), a molecular feature previously supported by fluorescence in situ hybridization (FISH) evidence showing a modest proportion of Vmat2/VGLUT2 double-positive cells in the medial VTA [43]. Further cellular communication analysis revealed that it is the neuronal subpopulation that communicated the most with dopaminergic neurons in terms of the number and strength of signaling pathways. These findings align with the glutamatergic neurons identified by Zell et al. [18] that can promote DA release to some extent.

Furthermore, *Kitl*, which expressed highly in Cluster 5 of KO_S group, also showed significant differences at the protein level between the KO_S group and the WT_S group. Kit ligand (Kitl), a single-pass transmembrane protein, contains an extracellular domain essential for facilitating Kit dimerization and downstream kinase activation. Earlier research has reported the coexpression of KL and its receptor Kit within selectively connected neuronal populations [52,53]. More recently, Zaman et al. [54] established the functional significance of the cKit–Kitl complex in supporting synaptic formation and maintenance among molecular layer interneurons that interact with cerebellar Purkinje cells. Interestingly, Purkinje cells have been implicated in reward delivery [55]. The observed up-regulation of KIT and KITL may also be linked to Wnt signaling [56].

The Wnt signaling pathway not only plays a critical role in the development of various neuronal functions such as neuronal proliferation, survival, migration, and differentiation, but also influences synaptic plasticity regulation, which is highly related to learning and memory, as well as reward responses and addictive behaviors [57,58]. Importantly, the degradation machinery of the canonical Wnt pathway can regulate the stability of p120-catenin family proteins—such as ARVCF-catenin and δ-catenin—via mechanisms that are also involved in the control of β-catenin [59]. It has been reported that microinjection of Wnt inhibitor IWP-2 into the NAc of rats impaired the acquisition of CPP [60]. The enhancement of the Wnt/β-catenin signaling pathway promotes the proliferation of midbrain dopaminergic neural precursor cells [61], and blockade of it would impair the locomotion in response to increased DA release [62].

It is known that the nicotinic acetylcholine receptors (nAChRs) are involved in the pathogenesis of various neurological and psychiatric disorders, such as Alzheimer’s disease, depression, attention deficit/hyperactivity disorder, and drug addiction [63–65]. Specifically, nicotine modulates neuronal network activity via binding to nAChRs with varying affinities and alters the expression of various nAChR subtypes, which eventually lead to the development of ND [66]. The α7*-nAChRs regulate the function of the nervous system in different ways, participating in various physiological functions such as cognition, learning and memory, and emotional behavior changes [67]. Our immunofluorescence analysis revealed that *Arvcf*-KO down-regulated Chrna7 expression in the mice VTA, an effect that was reversible upon treatment with the Wnt/β-catenin pathway activator TWS119. Similarly, the use of Wnt activator to treat emotional disorders associated with the copy number replication of *CHRNA7* partially reversed the impaired inhibitory interneuron migration in the neuromorphic system [68]. The immunofluorescence results also demonstrated that Chrna7 within mice VTA reached its higher levels following nicotine treatment, which is consistent with the earlier observations at the mRNA level [69]. Liu et al. [70,71] demonstrated that intraperitoneal administration of methyllycaconitine citrate, a selective antagonist of α7*-nAChRs, effectively suppressed context-induced nicotine-seeking behavior in rats in a dose-dependent manner. Notably, these authors further reported that α4β2* nAChRs did not exhibit a similar regulatory role in this behavior [70–72]. The α7*-nAChRs in VTA are known to play a role in promoting glutamatergic transmission [73] and even regulating the discharge pattern of DA neurons [74,75]. Additionally, it was demonstrated their significance in the facilitation of nicotine-triggered long-term potentiation (LTP) within the VTA [76,77], a regulatory ability on VTA neural plasticity that may share downstream plasticity pathways with the remarkable consciousness arousal effects observed upon ultrasound stimulation of the VTA in mice with traumatic brain injury [78].

It was documented that increased glutamate in the VTA is necessary for driving phasic firing associated with reward-seeking behavior, activating dopaminergic projections from VTA to NAc [20], and nicotine stimulation [79]. By integrating multi-omics profiles—including single-cell transcriptomics, metabolite detection, and neurology proteomics—we consistently observed damages in glutamatergic properties following *Arvcf* knockout. Metabolite detection revealed a significant reduction in glutamate levels within the VTA of *Arvcf*-KO mice (*P* = 0.0149). At the single-neuron level, coexpression proportion analysis in neuronal Cluster 5 demonstrated a marked decrease in the proportion of glutamatergic markers in *Arvcf*-KO mice (*P* = 0.003762). Supporting these findings, both transcriptomic MCODE and proteomic GSEA results indicated stronger enrichment of glutamatergic synapse pathways in the WT_S group compared to the KO_S group. Furthermore, in addition to Cluster 5, other classical *Slc17a6*+ glutamatergic neuronal subpopulations within the VTA also exhibited enhanced glutamate metabolic cycling following nicotine treatment in WT mice, whereas this response was absent in *Arvcf*-KO mice. This broader response highlights the generalized importance of *Slc17a6*+ neurons in nicotine-induced neuroadaptation. Consistent with these observations, 2-way ANOVA further confirmed a significant interaction effect between genotype and nicotine treatment, with the resulting gene set being directly enriched in biological processes related to response to nicotine and ethanol. These findings collectively highlight a molecular link between *Arvcf* deficiency and reward-related behavioral plasticity, underscoring the critical role of *Slc17a6*+ glutamatergic neurons in this process. These results may explain why *Arvcf*-KO mice had poorer CPP of nicotine or alcohol in our recent work [27–29].

These findings, primarily derived from studies in male mice using nicotine reward based on the *Arvcf*-KO model, offer one perspective on a highly complex brain reward mechanism. However, the current work does not address potential sex-dependent differences in response. Despite this, our findings still allow us to propose a molecular network mediated by *Arvcf* (Fig. S9). By integrating key findings from proteomic and transcriptomic analysis, we propose a PPI model of nicotine-induced reward processing, which links *Arvcf* along with its interaction partners (N-cadherin and *Cdh2*) found by our group [29], glutamatergic–dopaminergic signaling, and behavioral output. Of course, this hypothetical framework would require further experimental validation and in-depth exploration to fully elucidate the mechanistic link between *Arvcf* and Wnt signaling and the roles of *Arvcf* in reward circuitry.

Although the proportion of dopaminergic neurons observed is similar to previous reported studies [21,34], it should be acknowledged that there may be underrepresentation of this type of neuron in existing datasets. Moreover, despite our effort to exclude the SN and IPN based on marker gene expression, it is still possible that our dataset includes some non-VTA neurons from adjacent regions, for which no definitive markers are available yet to guide their exclusion. Additionally, our current research mainly elucidates the important role of VTA *Slc17a6*+ glutamatergic neurons in reward processing, and the potential interaction between these neurons’ dual functions in reward and aversion (via LHb projection) [80] remains an important area for future investigation. A comprehensive understanding of how these seemingly opposite functions are coordinated at the circuit and molecular levels is crucial for revealing the complex neural regulatory mechanisms behind motivational behavior.

## Conclusion

In conclusion, at the single-cell level, we first found that *Arvcf* was densely expressed in VTA neurons, particularly in the GLU>DA combinatorial neurons. These neurons may engage in cell communication with dopaminergic neurons, a process in which the Wnt signaling pathway could be involved. Further, we highlighted the important role of glutamatergic properties in reward learning and ND. Taken together, our findings provide a new perspective for the prevention and intervention of reward-seeking behaviors.

## Methods

### Generation of *Arvcf* knockout animals

The mice used in the study were 8- to 10-week-old males with a C57BL/6 background. C57BL/6 WT mice were purchased from GemPharmatech Co. Ltd. (Nanjing, China). The *Arvcf*-KO mice were obtained from Dr. Yang [27], which were generated using the CRISPR/Cas9 system at GemPharmatech Co. Ltd. (Nanjing, China). Knockout of the *Arvcf* gene was confirmed by quantitative real-time polymerase chain reaction (qRT-PCR) and Western blot. The primers used for qRT-PCR in this study were 5′-GCAGAACCATGAAGCTCAGGGA-3′ (forward) and 5′-CATACCTTAGTCTCAGTCCGCCG-3′ (reverse). The study was approved by the Animal Care and Use Committee of the First Affiliated Hospital of Zhejiang University (Approval no. 2023-596).

### Collection of VTA tissue from adult mice

Both the *Arvcf*-KO and WT mice were from the same litter. After euthanasia by using 100 mg/kg sodium pentobarbital, the brain tissues of each mouse were rapidly isolated under ice and cleaned with phosphate-buffered saline [29]. Then, the brain tissues were placed on a shock slicer to cut the brain tissues into 300-μm-thick coronal slices. Combined with the Allen Brain Atlas (https://atlas.brain-map.org/), the VTA brain region corresponding to Bregma (between –2.9 and –3.7 mm) was carefully dissected under a dissection microscope (Ivesta 3, Leica) and stored at –80°C until use.

### snRNA-seq and data processing

Both *Arvcf*-KO and WT mice were divided into nicotine-treated or saline-control groups with 3 animals per group. Nicotine in the nicotine-treated group was administered subcutaneously at a dose of 0.5 mg/kg/day for 7 days validated by the CPP test [27]. At the end of experiment, we collected VTA tissues and conducted single-nucleus dissociation as previously described [21]. After single-nucleus dissociation, cDNA library was constructed using a 10× Genomics Chromium Single-Cell 3′ kit (v3) and then sequenced at a minimum depth of 20,000 reads per cell on the Illumina NovaSeq 6000 Sequencing System (Illumina Inc., San Diego, USA). The Illumina bcl2fastq (v2.20) software was used to convert the sequencing data into FASTQ format.

Raw sequencing data were aligned to the mouse reference genome (v. mm10) using CellRanger software (v7.1.0) to generate raw gene expression matrices for each sample. Scrublet (v0.2.3) [81] was used to predict and exclude doublet cells per sample with default settings (https://github.com/AllonKleinLab/scrublet) in Python (v3.9). Then, we used Seurat R package (v5) [82] for all downstream analyses. Cells with <300 genes or >6,000 genes, nCount_RNA <500, and >1% mitochondrial genes were all filtered out. Batch effects were corrected through PCA with subsequent adjustment using Harmony [83]. Finally, a total of 96,240 filtered cells was selected for subsequent analysis.

The gene expression datasets were transformed by LogNormalize() and normalized using ScaleData(). PCA was applied to identify important principal components, and a total of 16 components determined by ElbowPlot() were selected for uniform manifold approximation and projection (UMAP) analysis with Leiden clustering algorithm [84]. We used the Local Inverse Simpson’s Index to ensure good integration of 12 samples. LISI-batch was calculated using lisi R package (v1.0) with default parameters. DEGs identified by FindAllMarkers() with parameters “min.pct = 0.25” and “thresh.use = 0.25” were compared to previous identified marker genes for the determination of the cell type of each cluster. Resulting *P* values were adjusted using the Bonferroni correction based on the number of genes identified in each cluster. The CellMarker2.0 database (http://117.50.127.228/CellMarker/) was used to perform cell type annotation. In addition, we used CellCycleScoring() to further identify the neuronal population of our interest. Then, we used scCODA (https://github.com/theislab/scCODA) to statistically confirm proportional differences in cell-type abundance caused by *Arvcf*-KO and nicotine treatment at an FDR level of 0.05.

### Neuronal reclustering of single-cell transcriptomes

A total of 25,924 neurons was further clustered and annotated. Data from each sample were then integrated with IntegrateLayers() with parameters “method = ‘RPCAIntegration’” and “reference = which(levels(seurat_obj$group)==“WT_S”)”. The clustering resolution was determined using the following dual-strategy approach. We first calculated the modularity *Q* value across a range of resolutions (0.1 to 1.0 at an increment of 0.1) and selected the value at the inflection point where the modularity *Q* value increase began to plateau, which corresponded to a resolution of 0.2. This choice was further validated by iteratively increasing the resolution until a cluster no longer contained any marker gene with an AUC > 0.6 [85], confirming that 0.2 was the highest resolution avoiding biologically meaningless subdivisions. DEGs between the KO_S group and the WT_S group were identified by FindMarkers() with Wilcoxon method.

### Calculation of coexpression proportion of neuronal markers

To investigate cells with the potential to synthesize and transport multiple neurotransmitters, we first identified cells that coexpressed 2 genes associated with different neurotransmitters [21]. This was done by extracting a count matrix, which contained log-normalized gene counts for *Slc6a1* (solute carrier family 6 member 1), *Slc32a1* (solute carrier family 32 member 1), *Gad1* (glutamate decarboxylase 1), *Gad2* (glutamate decarboxylase 2), *Ddc* (dopa decarboxylase), *Th* (tyrosine hydroxylase), *Slc18a2* (solute carrier family 18), *Slc6a3* (solute carrier family 6), *Slc17a6* (solute carrier family 17 member 6), *Slc17a7* (solute carrier family 17 member 7), and *Grm2* (glutamate metabotropic receptor 2) for each neuronal subcluster. We then determined the number of cells with log-normalized count values greater than 0 for 2 of the above genes and divided this by the total number of cells within the cluster. The resulting value represented the proportion of cells within a specific neuronal subtype that coexpressed 2 genes involved in different neurotransmitter systems. A neuronal cluster was classified as “combinatorial” if the coexpression proportion of marker genes from distinct neurotransmitter systems exceeded 20% [21].

### Gene set enrichment analysis

GO BP enrichment analysis was performed by GSEA v.4.3 (http://www.broadinstitute.org/gsea/) using MSigDB geneset c5 v2023.1, and NES was used to estimate GSEA enrichment. The significance of enrichment was measured as *P* < 0.05.

### Analysis of intercellular communication with dopaminergic neurons

To explore the neuron subpopulations that can signal to dopaminergic neurons as well as the mechanism of action, we combined CellChat [86] and NicheNet [87] for cell–cell communication analysis. Firstly, we used ChellChat [86] to identify the number and strength of ligand–receptor pathways that can signal to dopaminergic neurons from each neuronal subpopulation in the WT_S group. Then, we used rankNet() to identify and visualize conserved and specific signaling pathways between the WT_S group and the KO_S group by comparing the information flow of each significant signaling pathway with *P* < 0.05. The official workflow of CellChat analysis is detailed at https://github.com/sqjin/CellChat. Furthermore, we used Differential NicheNet (https://github.com/saeyslab/nichenetr), an extension of the default NicheNet algorithm [87], to predict the active ligand–receptor junctions and targets that were differentially expressed or activated between the KO_S group and the WT_S group.

### Single-cell flux estimation analysis

The normalized count matrix of the subpopulation of *Slc17a6*-positive glutamatergic neurons (Clusters 0, 5, 6, 8, 10, 16, 17, and 18) was used as input for the single-cell flux estimation analysis (scFEA) software [88], a computational tool designed to infer metabolic flux states from transcriptomic data. The resulting output consisted of reaction fluxes estimation for each cell.

### Two-way ANOVA

For the subpopulation of *Slc17a6*-positive glutamatergic neurons (Clusters 0, 5, 6, 8, 10, 16, 17, and 18), we first performed pseudo-bulk transformation for each group (*n* = 3/group) using the Muscat R package. Then, we performed a 2-way ANOVA using the aov() function in R and organized the results with the broom R package (v1.0.4) to determine the interaction effect between genotype (*Arvcf-*KO) and nicotine treatment. Partial eta squared > 0.06 (medium effect size) with *P* < 0.05 was considered statistically significant [89].

### Construction of a PPI network for genes related to *Arvcf-*mediated reward learning

We employed the STRING database (https://string-db.org/) to construct an interaction network associated with *Arvcf*-mediated reward learning, using *Arvcf* itself along with its known interaction partners—N-cadherin (*Cdh2*) [29], DAPs, predicted ligand–receptor pairs from cell communication analysis, and relevant target genes as inputs. The core PPI data were then imported into Cytoscape software (v3.8.0) for network construction.

### Olink proteomics analysis

A total of 16 WT and *Arvcf*-KO 8- to 10-week control-treated mice were divided into 2 groups (*n* = 8/group), after which VTA tissues were collected from all mice using the method mentioned above. Proteomic profiling was conducted with the Olink Target 96 Neurology panel according to the manufacturer’s protocol. Normalized protein expression (NPX) values on a log_2_ scale were generated through the Olink NPX Manager software (Olink, Uppsala, Sweden). DAPs were identified using the OlinkAnalyze R package (v4.2.0), applying a significance threshold of *P* < 0.05.

### Detection of neurotransmitters by high-performance liquid chromatography–mass spectrometry

Using high-performance liquid chromatography–mass spectrometry, we analyzed glutamate levels in VTA tissue extracted from *Arvcf*-KO and WT mice (*n* = 7/group, methods as above). We prepared the tissue homogenate and centrifuged the homogenate to take the supernatant; the supernatant was chromatographically separated by the ACQUITY UPLC I-Class system (Waters Co., Milford, MA, USA). The constituents were then detected by the Xevo TQ-XS tandem quadrupole mass spectrometry system (Waters Co., Milford, MA, USA) with an ion source voltage of 3.0 kV and a temperature of 150 °C, a desolvation temperature of 400 °C, a desolvation gas flow rate of 800 l/h, and a conical pore gas flow rate of 150 l/h. Quantitative analysis was performed by measuring peak areas in TargetLynx software and deriving concentrations from standard curves.

### Wnt/β-catenin pathway activator treatment

TWS119 (MedChemExpress, HY-10590), dissolved in 1% dimethyl sulfoxide (DMSO), was intraperitoneally administered once daily to *Arvcf*-KO mice for 14 days, following an established protocol [90]. Injections were performed aseptically in accordance with standard procedures to minimize the risk of complications such as peritonitis. The dosage of 10 mg/kg was chosen based on previous experimental data [42].

### Immunofluorescence staining

VTA tissue samples were fixed in 4% paraformaldehyde (PFA), dehydrated, and embedded in paraffin blocks. Sections were cut at 10 μm thickness using a Microtome (Leica HistoCoreBIOCUT) and mounted on glass slides. For single labeling of CHRNA7, sections were incubated with rabbit anti-Chrna7 (1:50; ABclonal, A1588). For triple labeling, multiplex fluorescence was performed using TSA with Opal fluorophores (Akoya Biosciences) following sequential staining with rabbit anti-Th (1:200; ProteinTech, 25859-1-AP), rabbit anti-VGLUT2 (1:50; ProteinTech, 29209-1-AP), and rabbit anti-Vmat2 (1:50; ProteinTech, 20873-1-AP). Between each cycle, heat-mediated antibody stripping was performed. Images were acquired using a confocal microscope (NIKON ECLIPSE C1). Mean fluorescence density was quantified in defined regions of interest using the ImageJ software.

## Data Availability

Sequencing data have been deposited in the Genome Sequence Archive under accession number CRA016632 (https://ngdc.cncb.ac.cn/gsub/). Further details can be obtained from the corresponding author.
