# Supplementary material for: Single-Cell Profiling Identifies Reward Behavior-Related Neurons and Alterations in the Ventral Tegmental Area Based on Arvcf-Knockout Mouse Model
Source: Research (Wash D C). 2025 Dec 10;8:1030. doi: 10.34133/research.1030 (PMC12695470; doi:10.34133/research.1030)
Supplement: Supplementary 1 — Figs. S1 to S9 Tables S1 to S9 [file research.1030.f1.zip › Supplementary Information(Figure Captions).docx]

**Supplementary Figures**

Figure S1. (A) Strategy of constructing *Arvcf*-KO mice by deleting exons 4-10. Abbreviations: ATG = start codon, TAG = stop codon. (B) Sequences of Arvcf wild-type (*Arvcf*-WT) mice and *Arvcf*-KO mice by deleting 5805 bp among exons 4-10 [27]. (C) qPCR result of Arvcf mRNA normalized to reference gene Gapdh in the WT and *Arvcf*-KO groups (n=3/group), *** p < 0.001 between indicated conditions. (D) Western blotting of Arvcf and β-actin in WT and *Arvcf*-KO mice. β-actin is a reference gene.

Figure S2. (A) Scatter plot indicating our snRNA-seq data usability. Pearson’s correlations on the top show that nCount_RNA is not related to the percentage of mitochondrial gene expression (percent.mt) (left), and nCount_RNA is strongly associated with nFeature_RNA (right). (B) Dot plot indicates that the standard deviation stabilizes after PC = 16, so we choose it as the parameter. (C) Before QC filtration, violin plots indicating three QC indicators: nFeature_RNA, nCount_RNA and percent.mt. (D) UMAP displaying all cells after quality control (QC) filtration of 12 mice. (E) Ridge plot showing the distribution of Local Inverse Simpson’s Index (LISI) for each sample. The LISI value is closer to 1, it indicates poorer cell mixing; the LISI value is closer to the sample size (n = 12), the better the cell mixing. (F) UMAP showing the phase of each cell. The cell cycle of the neuronal population is mostly in the G1 and S phases. (G) UMAPs of different groups.

Figure S3. Bar plots show log2FC in cell type abundance (KO_S vs WT_S, WT_N vs WT_S, and KO_N vs KO_S), calculated by scCODA. *: significance at an FDR level of 0.05.

Figure S4. (A) Dot-line plot shows Modularity Q value in each resolution of neuronal subpopulations. (B) Dot-line plot shows the percentage of clusters which AUC > 0.6 in each resolution of neuronal subpopulations.

Figure S5. Heatmaps depicting the overall co-expression proportion patterns of neurotransmitter-related genes (GABA, DA, glutamate) involved in synthesis, release and transport across each individual cluster (A-C: Clusters 3, 8 and 18, GLU>GABA; D: Cluster 0, GLU+GABA; E: Cluster 0, GLU>DA).

Figure S6. (A) UMAP plots showing gene expression level of some dopaminergic related genes in DA neurons. (B) The co-expression (yellow dots) of Sox6 (red dots) and Aldh1a1 (green dots) in DA neurons. (C) UMAP plots showing gene expression level of interpeduncular nucleus (IPN) markers. (D) Dot plot displaying Grm2 has a higher expression level in Clusters 3 and 8; UMAP plot displaying Grm5 is commonly expressed in all neurons.

Figure S7. (A) The scaled gene expression level of top 50 ligands by NicheNet. (B) Top 50 ligands’ minimum log_2_FC (LFC) in sender cells (Cluster 5) and receiver cells (Cluster 13), each row is a top ligand-receptor pair computed using the minimum LFC of ligand, with each ligand exhibiting top 2 receptors.

Figure S8. UMAP plots displays the gene expression level of Slc17a6 and Slc17a7.

Figure S9. The protein-protein interaction (PPI) network related to Arvcf mediated reward learning predicted by the STRING database. (A) The predicted PPI interaction network predicted by the “more” function. The red box indicates the core network. (B) The core network and predicted downstream functional pathways. The input features are marked in dark pink, and the predicted proteins are marked in light pink.
