# Supplementary material for: Single-Cell Profiling Identifies Reward Behavior-Related Neurons and Alterations in the Ventral Tegmental Area Based on Arvcf-Knockout Mouse Model
Source: Research (Wash D C). 2025 Dec 10;8:1030. doi: 10.34133/research.1030 (PMC12695470; doi:10.34133/research.1030)
Supplement: Supplementary 1 — Figs. S1 to S9 Tables S1 to S9 [file research.1030.f1.zip › SupplementaryFigures.pdf]

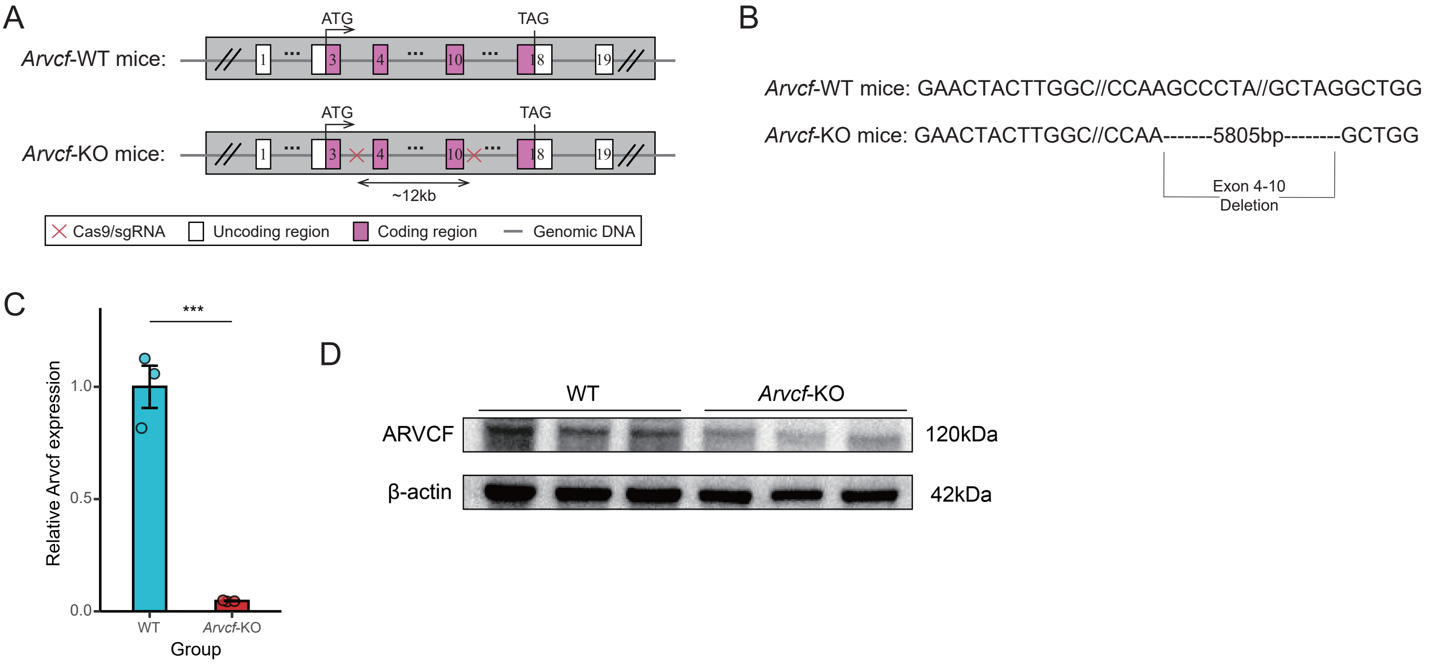
A
Arvcf-WT mice:
ATG
TAG
1
3
4
10
18
19
Arvcf-KO mice:
~12kb
Cas9/sgRNA
Uncoding region
Coding region
Genomic DNA
B
Arvcf-WT mice: GAACTACTTGGC//CCAAGCCCTA//GCTAGGCTGG
Arvcf-KO mice: GAACTACTTGGC//CCAA-------5805bp--------GCTGG
Exon 4-10
Deletion
C
***
Relative Arvcf expression
1.0
0.5
0.0
WT
Arvcf-KO
Group
D
WT
Arvcf-KO
ARVCF
120kDa
β-actin
42kDa

Figure S1

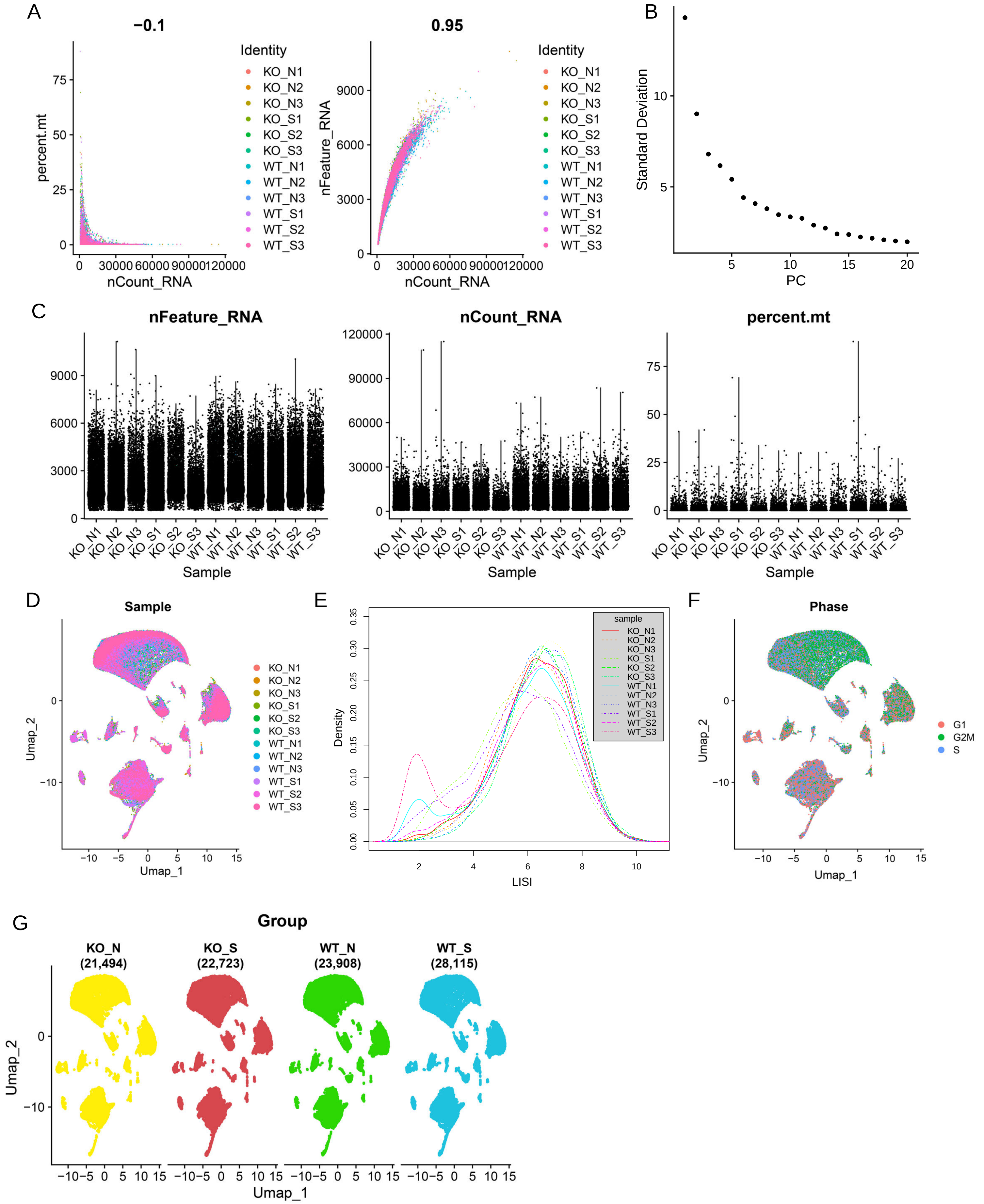
A
−0.1
0.95
Identity
KO_N1
KO_N2
KO_N3
KO_S1
KO_S2
KO_S3
WT_N1
WT_N2
WT_N3
WT_S1
WT_S2
WT_S3
percent.mt
nCount_RNA
nFeature_RNA
B
Standard Deviation
PC
C
nFeature_RNA
nCount_RNA
percent.mt
Sample
D
Sample
Umap_2
Umap_1
E
sample
Density
LISI
F
Phase
G1
G2M
S
G
Group
KO_N (21,494)
KO_S (22,723)
WT_N (23,908)
WT_S (28,115)

Figure S2

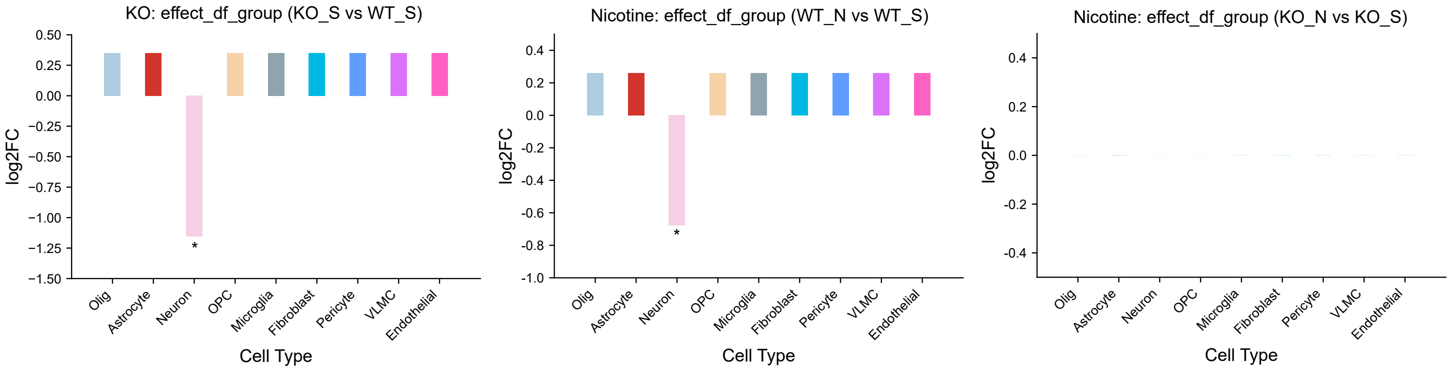
KO: effect_df_group (KO_S vs WT_S)
Nicotine: effect_df_group (WT_N vs WT_S)
Nicotine: effect_df_group (KO_N vs KO_S)
log2FC
Cell Type
Olig
Astrocyte
Neuron
OPC
Microglia
Fibroblast
Pericyte
VLMC
Endothelial
*

Figure S3

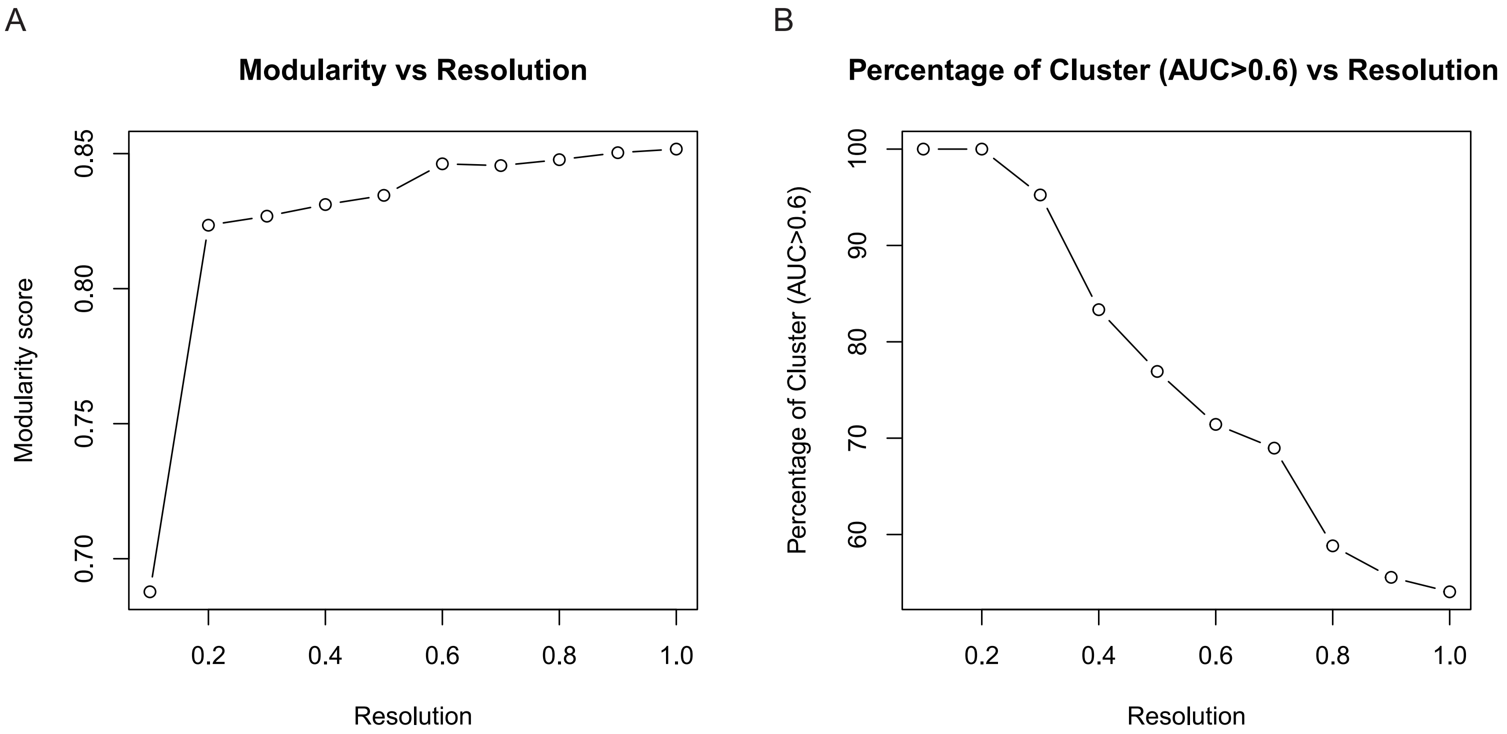
A
Modularity vs Resolution
Modularity score
0.70
0.75
0.80
0.85
0.2
0.4
0.6
0.8
1.0
Resolution
B
Percentage of Cluster (AUC>0.6) vs Resolution
Percentage of Cluster (AUC>0.6)
60
70
80
90
100
0.2
0.4
0.6
0.8
1.0
Resolution

Figure S4

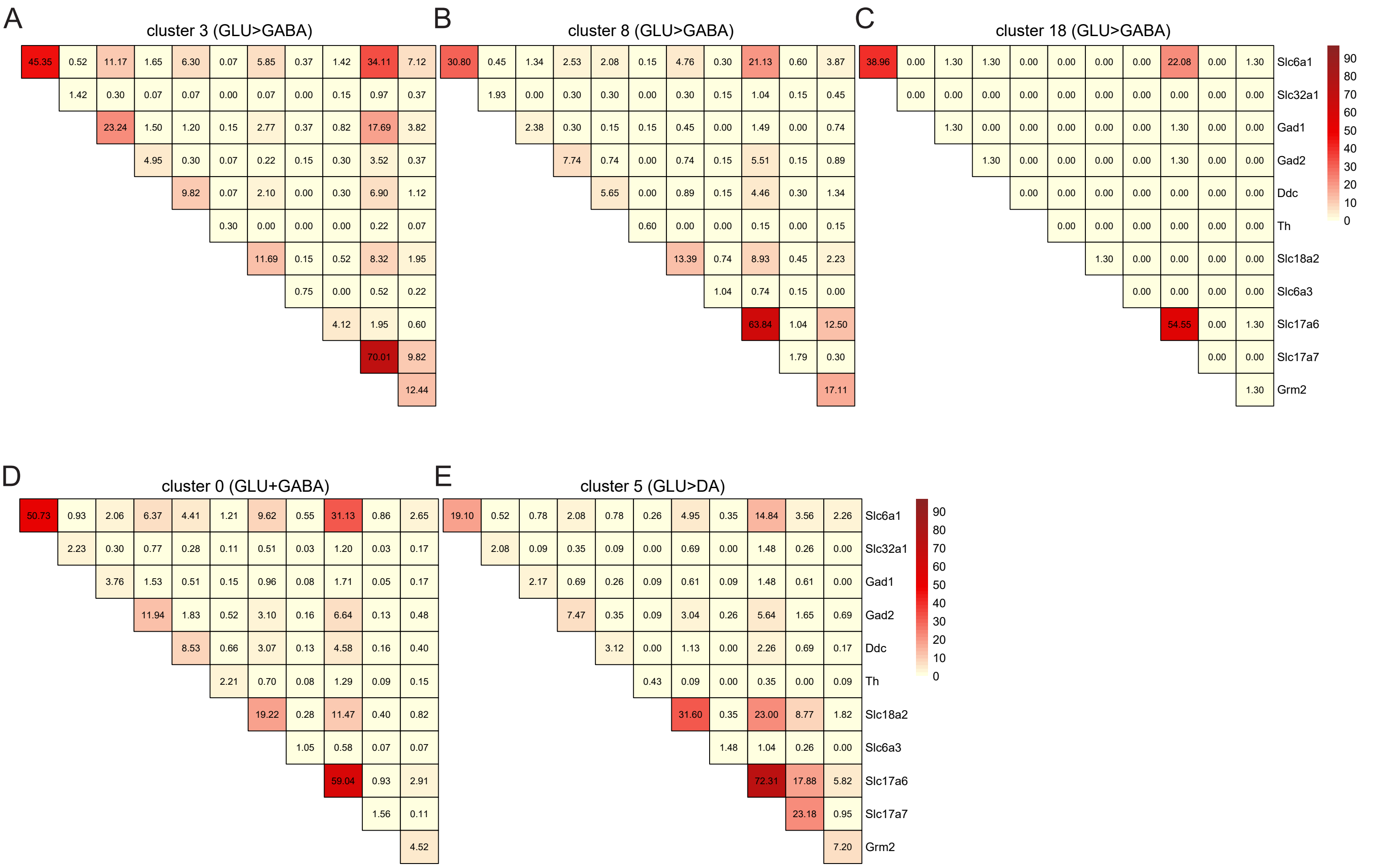
A
cluster 3 (GLU>GABA)
45.35 0.52 11.17 1.65 6.30 0.07 5.85 0.37 1.42 34.11 7.12
1.42 0.30 0.07 0.07 0.00 0.07 0.00 0.15 0.97 0.37
23.24 1.50 1.20 0.15 2.77 0.37 0.82 17.69 3.82
4.95 0.30 0.07 0.22 0.15 0.30 3.52 0.37
9.82 0.07 2.10 0.00 0.30 6.90 1.12
0.30 0.00 0.00 0.00 0.22 0.07
11.69 0.15 0.52 8.32 1.95
0.75 0.00 0.52 0.22
4.12 1.95 0.60
70.01 9.82
12.44
B
cluster 8 (GLU>GABA)
30.80 0.45 1.34 2.53 2.08 0.15 4.76 0.30 21.13 0.60 3.87
1.93 0.00 0.30 0.30 0.00 0.30 0.15 1.04 0.15 0.45
2.38 0.30 0.15 0.15 0.45 0.00 1.49 0.00 0.74
7.74 0.74 0.00 0.74 0.15 5.51 0.15 0.89
5.65 0.00 0.89 0.15 4.46 0.30 1.34
0.60 0.00 0.00 0.15 0.00 0.15
13.39 0.74 8.93 0.45 2.23
1.04 0.74 0.15 0.00
63.84 1.04 12.50
1.79 0.30
17.11
C
cluster 18 (GLU>GABA)
38.96 0.00 1.30 1.30 0.00 0.00 0.00 0.00 22.08 0.00 1.30
0.00 0.00 0.00 0.00 0.00 0.00 0.00 0.00 0.00 0.00
1.30 0.00 0.00 0.00 0.00 0.00 1.30 0.00 0.00
1.30 0.00 0.00 0.00 0.00 1.30 0.00 0.00
0.00 0.00 0.00 0.00 0.00 0.00 0.00
0.00 0.00 0.00 0.00 0.00 0.00
1.30 0.00 0.00 0.00 0.00
0.00 0.00 0.00 0.00
54.55 0.00 1.30
0.00 0.00
1.30
Slc6a1
Slc32a1
Gad1
Gad2
Ddc
Th
Slc18a2
Slc6a3
Slc17a6
Slc17a7
Grm2
90
80
70
60
50
40
30
20
10
0
D
cluster 0 (GLU+GABA)
50.73 0.93 2.06 6.37 4.41 1.21 9.62 0.55 31.13 0.86 2.65
2.23 0.30 0.77 0.28 0.11 0.51 0.03 1.20 0.03 0.17
3.76 1.53 0.51 0.15 0.96 0.08 1.71 0.05 0.17
11.94 1.83 0.52 3.10 0.16 6.64 0.13 0.48
8.53 0.66 3.07 0.13 4.58 0.16 0.40
2.21 0.70 0.08 1.29 0.09 0.15
19.22 0.28 11.47 0.40 0.82
1.05 0.58 0.07 0.07
59.04 0.93 2.91
1.56 0.11
4.52
E
cluster 5 (GLU>DA)
19.10 0.52 0.78 2.08 0.78 0.26 4.95 0.35 14.84 3.56 2.26
2.08 0.09 0.35 0.09 0.00 0.69 0.00 1.48 0.26 0.00
2.17 0.69 0.26 0.09 0.61 0.09 1.48 0.61 0.00
7.47 0.35 0.09 3.04 0.26 5.64 1.65 0.69
3.12 0.00 1.13 0.00 2.26 0.69 0.17
0.43 0.09 0.00 0.35 0.00 0.09
31.60 0.35 23.00 8.77 1.82
1.48 1.04 0.26 0.00
72.31 17.88 5.82
23.18 0.95
7.20
Slc6a1
Slc32a1
Gad1
Gad2
Ddc
Th
Slc18a2
Slc6a3
Slc17a6
Slc17a7
Grm2
90
80
70
60
50
40
30
20
10
0

Figure S5

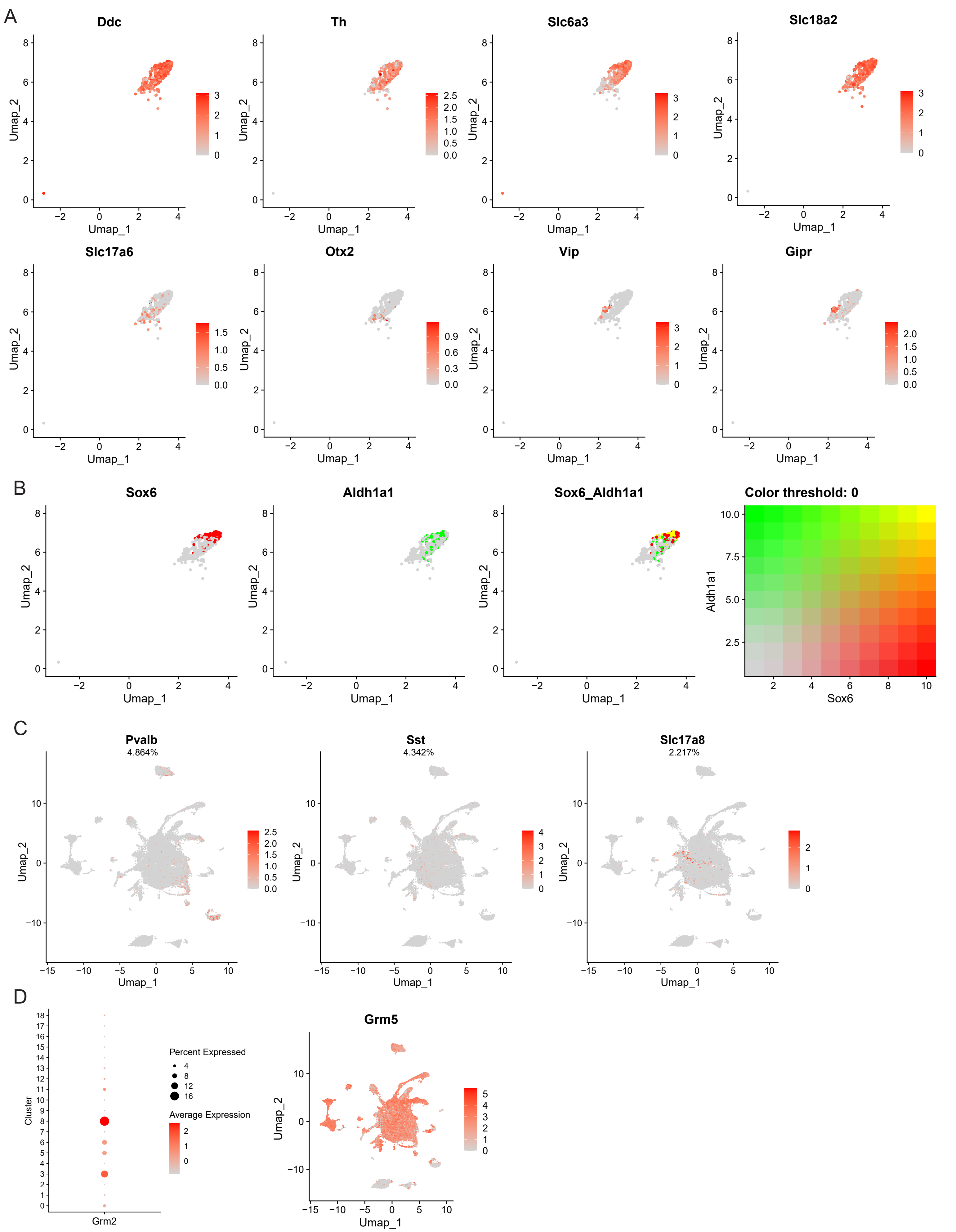
A
Ddc
Th
Slc6a3
Slc18a2
Slc17a6
Otx2
Vip
Gipr
Umap_1
Umap_2
B
Sox6
Aldh1a1
Sox6_Aldh1a1
Color threshold: 0
C
Pvalb
4.864%
Sst
4.342%
Slc17a8
2.217%
D
Percent Expressed
Average Expression
Cluster
Grm2
Grm5

Figure S6

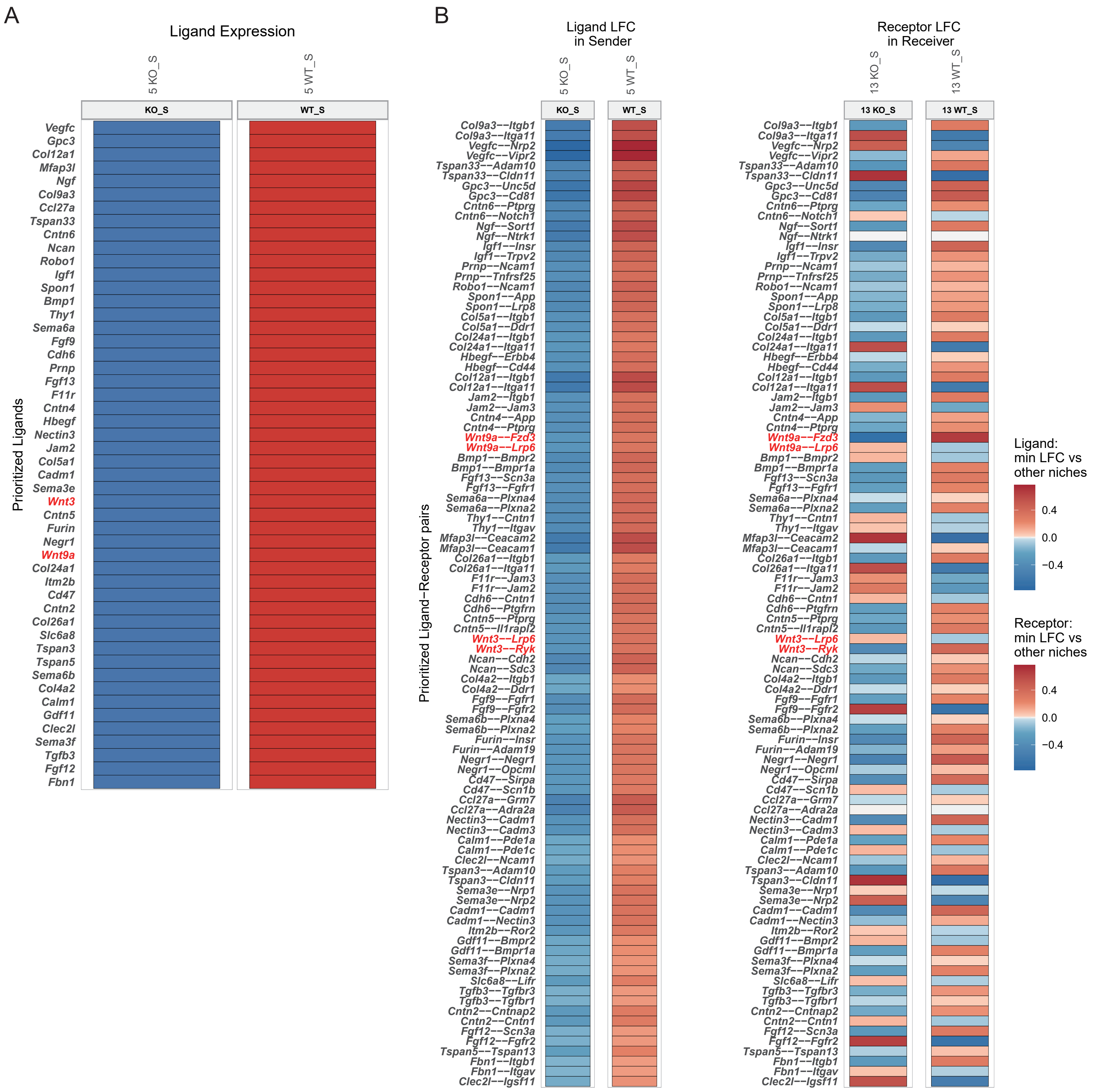
A
Ligand Expression
5 KO_S
5 WT_S
KO_S
WT_S
Prioritized Ligands
Vegfc
Gpc3
Col12a1
Mfap3l
Ngf
Col9a3
Ccl27a
Tspan33
Cntn6
Ncan
Robo1
Igf1
Spon1
Bmp1
Thy1
Sema6a
Fgf9
Cdh6
Prnp
Fgf13
F11r
Cntn4
Hbegf
Nectin3
Jam2
Col5a1
Cadm1
Sema3e
Wnt3
Cntn5
Furin
Negr1
Wnt9a
Col24a1
Itm2b
Cd47
Cntn2
Col26a1
Slc6a8
Tspan3
Tspan5
Sema6b
Col4a2
Calm1
Gdf11
Clec2l
Sema3f
Tgfb3
Fgf12
Fbn1
B
Ligand LFC in Sender
Receptor LFC in Receiver
5 KO_S
5 WT_S
13 KO_S
13 WT_S
KO_S
WT_S
13 KO_S
13 WT_S
Prioritized Ligand−Receptor pairs
Col9a3−−Itgb1
Col9a3−−Itga11
Vegfc−−Nrp2
Vegfc−−Vipr2
Tspan33−−Adam10
Tspan33−−Cldn11
Gpc3−−Unc5d
Gpc3−−Cd81
Cntn6−−Ptprg
Cntn6−−Notch1
Ngf−−Sort1
Ngf−−Ntrk1
Igf1−−Insr
Igf1−−Trpv2
Prnp−−Ncam1
Prnp−−Tnfrsf25
Robo1−−Ncam1
Spon1−−App
Spon1−−Lrp8
Col5a1−−Itgb1
Col5a1−−Ddr1
Col24a1−−Itgb1
Col24a1−−Itga11
Hbegf−−Erbb4
Hbegf−−Cd44
Col12a1−−Itgb1
Col12a1−−Itga11
Jam2−−Itgb1
Jam2−−Jam3
Cntn4−−App
Cntn4−−Ptprg
Wnt9a−−Fzd3
Wnt9a−−Lrp6
Bmp1−−Bmpr2
Bmp1−−Bmpr1a
Fgf13−−Scn3a
Fgf13−−Fgfr1
Sema6a−−Plxna4
Sema6a−−Plxna2
Thy1−−Cntn1
Thy1−−Itgav
Mfap3l−−Ceacam2
Mfap3l−−Ceacam1
Col26a1−−Itgb1
Col26a1−−Itga11
F11r−−Jam3
F11r−−Jam2
Cdh6−−Cntn1
Cdh6−−Ptgfrn
Cntn5−−Ptprg
Cntn5−−Il1rapl2
Wnt3−−Lrp6
Wnt3−−Ryk
Ncan−−Cdh2
Ncan−−Sdc3
Col4a2−−Itgb1
Col4a2−−Ddr1
Fgf9−−Fgfr1
Fgf9−−Fgfr2
Sema6b−−Plxna4
Sema6b−−Plxna2
Furin−−Insr
Furin−−Adam19
Negr1−−Negr1
Negr1−−Opcml
Cd47−−Sirpa
Cd47−−Scn1b
Ccl27a−−Grm7
Ccl27a−−Adra2a
Nectin3−−Cadm1
Nectin3−−Cadm3
Calm1−−Pde1a
Calm1−−Pde1c
Clec2l−−Ncam1
Tspan3−−Adam10
Tspan3−−Cldn11
Sema3e−−Nrp1
Sema3e−−Nrp2
Cadm1−−Cadm1
Cadm1−−Nectin3
Itm2b−−Ror2
Gdf11−−Bmpr2
Gdf11−−Bmpr1a
Sema3f−−Plxna4
Sema3f−−Plxna2
Slc6a8−−Lifr
Tgfb3−−Tgfbr3
Tgfb3−−Tgfbr1
Cntn2−−Cntnap2
Cntn2−−Cntn1
Fgf12−−Scn3a
Fgf12−−Fgfr2
Tspan5−−Tspan13
Fbn1−−Itgb1
Fbn1−−Itgav
Clec2l−−Igsf11
Ligand: min LFC vs other niches
0.4
0.0
−0.4
Receptor: min LFC vs other niches
0.4
0.0
−0.4

Figure S7

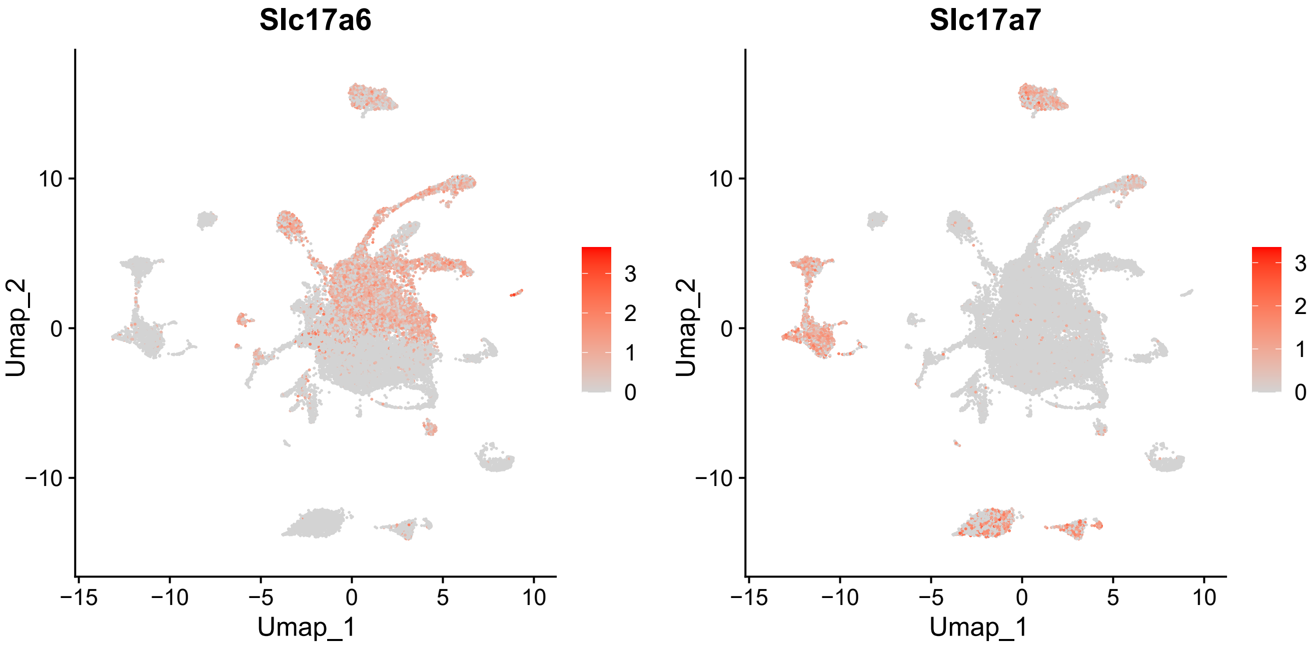

Figure S8

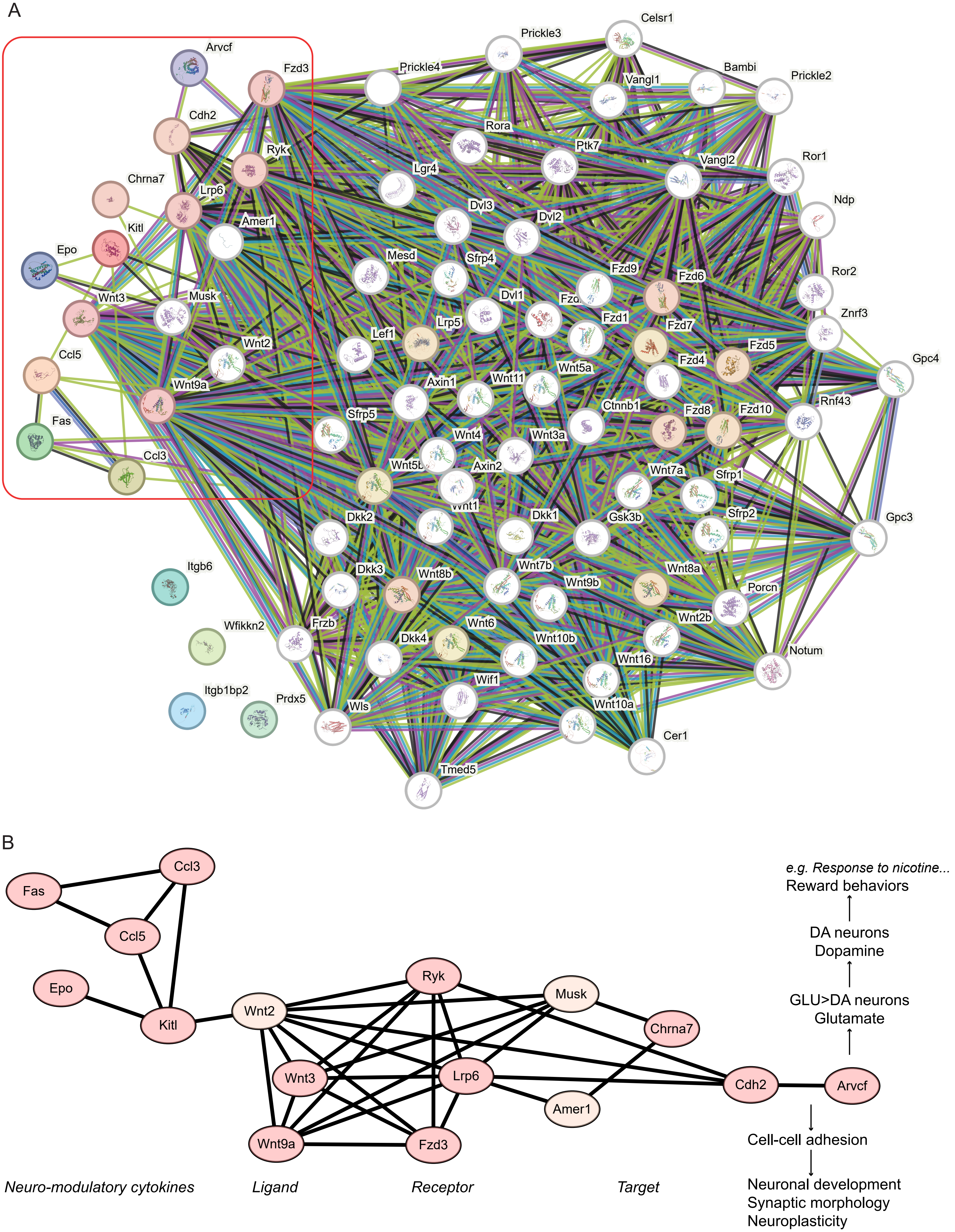
A
Arvcf
Fzd3
Cdh2
Ryk
Chrna7
Lrp6
Kitl
Amer1
Epo
Wnt3
Musk
Wnt2
Ccl5
Wnt9a
Fas
Ccl3
Prickle3
Celsr1
Prickle4
Vangl1
Bambi
Prickle2
Rora
Ptk7
Vangl2
Ror1
Lgr4
Dvl3
Dvl2
Ndp
Mesd
Sfrp4
Fzd9
Fzd6
Ror2
Dvl1
Fzd1
Fzd7
Znrf3
Lrp5
Lef1
Fzd5
Fzd4
Wnt5a
Gpc4
Axin1
Wnt11
Ctnnb1
Fzd8
Fzd10
Rnf43
Sfrp5
Wnt4
Wnt3a
Axin2
Wnt7a
Sfrp1
Dkk2
Dkk1
Gsk3b
Sfrp2
Gpc3
Itgb6
Dkk3
Wnt8b
Wnt7b
Wnt9b
Wnt8a
Porcn
Wfikkn2
Frzb
Wnt6
Dkk4
Wnt10b
Wnt2b
Notum
Wnt16
Wif1
Itgb1bp2
Prdx5
Wls
Wnt10a
Cer1
Tmed5
B
Fas
Ccl3
Ccl5
Epo
Kitl
Wnt2
Ryk
Musk
Chrna7
Wnt3
Lrp6
Cdh2
Arvcf
Amer1
Wnt9a
Fzd3
Neuro-modulatory cytokines
Ligand
Receptor
Target
e.g. Response to nicotine...
Reward behaviors
DA neurons
Dopamine
GLU>DA neurons
Glutamate
Cell-cell adhesion
Neuronal development
Synaptic morphology
Neuroplasticity

Figure S9
